# A proteomics study of rheumatoid arthritis patients on etanercept identifies putative biomarkers associated with clinical outcome measures

**DOI:** 10.1093/rheumatology/kead321

**Published:** 2023-06-30

**Authors:** Stephanie F Ling, Chuan Fu Yap, Nisha Nair, James Bluett, Ann W Morgan, John D Isaacs, Anthony G Wilson, Kimme L Hyrich, Anne Barton, Darren Plant

**Affiliations:** Centre for Genetics and Genomics Versus Arthritis, Centre for Musculoskeletal Research, The University of Manchester, Manchester, UK; NIHR Biomedical Research Centre Manchester, Manchester University NHS Foundation Trust, Manchester Academic Health Science Centre, Manchester, UK; Centre for Genetics and Genomics Versus Arthritis, Centre for Musculoskeletal Research, The University of Manchester, Manchester, UK; Centre for Genetics and Genomics Versus Arthritis, Centre for Musculoskeletal Research, The University of Manchester, Manchester, UK; NIHR Biomedical Research Centre Manchester, Manchester University NHS Foundation Trust, Manchester Academic Health Science Centre, Manchester, UK; Centre for Genetics and Genomics Versus Arthritis, Centre for Musculoskeletal Research, The University of Manchester, Manchester, UK; NIHR Biomedical Research Centre Manchester, Manchester University NHS Foundation Trust, Manchester Academic Health Science Centre, Manchester, UK; School of Medicine, University of Leeds, Leeds, UK; NIHR Leeds Biomedical Research Centre, Leeds Teaching Hospitals NHS Trust, Leeds, UK; NIHR In Vitro Diagnostic Co-operative, Leeds Teaching Hospitals NHS Trust, Leeds, UK; Translational and Clinical Research Institute, Newcastle University, Newcastle-upon-Tyne, UK; Musculoskeletal Unit, Newcastle-upon-Tyne Hospitals NHS Foundation Trust, Newcastle-upon-Tyne, UK; School of Medicine and Medical Science, Conway Institute, University College Dublin, Dublin, Ireland; NIHR Biomedical Research Centre Manchester, Manchester University NHS Foundation Trust, Manchester Academic Health Science Centre, Manchester, UK; Centre for Epidemiology Versus Arthritis, Centre for Musculoskeletal Research, The University of Manchester, Manchester, UK; Centre for Genetics and Genomics Versus Arthritis, Centre for Musculoskeletal Research, The University of Manchester, Manchester, UK; NIHR Biomedical Research Centre Manchester, Manchester University NHS Foundation Trust, Manchester Academic Health Science Centre, Manchester, UK; Centre for Genetics and Genomics Versus Arthritis, Centre for Musculoskeletal Research, The University of Manchester, Manchester, UK; NIHR Biomedical Research Centre Manchester, Manchester University NHS Foundation Trust, Manchester Academic Health Science Centre, Manchester, UK

**Keywords:** RA, biologics, anti-TNF, etanercept, treatment response, proteomics, genetics, biomarkers

## Abstract

**Objectives:**

Biologic DMARDs (bDMARDs) are widely used in patients with RA, but response to bDMARDs is heterogeneous. The objective of this work was to identify pretreatment proteomic biomarkers associated with RA clinical outcome measures in patients starting bDMARDs.

**Methods:**

Sequential window acquisition of all theoretical fragment ion spectra mass spectrometry (SWATH-MS) was used to generate spectral maps of sera from patients with RA before and after 3 months of treatment with the bDMARD etanercept. Protein levels were regressed against RA clinical outcome measures, i.e. 28-joint DAS (DAS28) and its subcomponents and DAS28 <2.6 (i.e. remission). The proteins with the strongest evidence for association were analysed in an independent, replication dataset. Finally, subnetwork analysis was carried out using the Disease Module Detection algorithm and biological plausibility of identified proteins was assessed by enrichment analysis.

**Results:**

A total of 180 patients with RA were included in the discovery dataset and 58 in the validation dataset from a UK-based prospective multicentre study. Ten individual proteins were found to be significantly associated with RA clinical outcome measures. The association of T-complex protein 1 subunit η with DAS28 remission was replicated in an independent cohort. Subnetwork analysis of the 10 proteins from the regression analysis identified the ontological theme, with the strongest associations being with acute phase and acute inflammatory responses.

**Conclusion:**

This longitudinal study of 180 patients with RA commencing etanercept has identified several putative protein biomarkers of treatment response to this drug, one of which was replicated in an independent cohort.

Rheumatology key messages

- There are currently no validated pretreatment protein biomarkers of RA treatment response.
- Identification of biomarkers of treatment response would allow more cost effective, informed decision making in RA treatment.
- We identified 10 protein biomarkers associated with clinical outcome measures in etanercept-treated patients with RA.

## Introduction

In patients with RA in whom conventional synthetic DMARD (csDMARD) therapy fails to control disease activity, treatment can be increased to more costly biologic DMARDs (bDMARDs) such as adalimumab and etanercept. However, treatment response to bDMARDs is not universal and, in up to 40% of these patients, inflammation remains inadequately controlled, either due to primary inefficacy or loss of response [1, 2]. Identification of reliable biomarkers predictive of response to these agents is a research priority, as predictive biomarkers would enable clinicians and patients to make informed therapy selection.

Multi-omics studies of biomarkers of treatment response are starting to accumulate in the RA literature and have typically focused on genetic, transcriptomic, metabolomic and lipidomic markers, but few have investigated proteomic biomarkers [3–5]. Proteins have many features that make them ideal potential biomarkers, given that proteins carry out diverse biological processes, interact with drugs and capture information on post-translational modifications. Furthermore, many proteins are stable and conventional assays commonly used in healthcare rely on protein technology (e.g. ELISAs), so translation of laboratory findings can be rapidly accelerated into clinical practice. Finally, recent technological advances in high-throughput methods mean that it is now possible to analyse large numbers of proteins in patient samples; e.g. sequential window acquisition of all theoretical fragment ion spectra mass spectrometry (SWATH-MS) is a high-coverage shotgun proteomics technique with near-comprehensive proteome cover and excellent dynamic range and reproducibility [6]. This enables the study of theoretically all spectra present in a sample, with a permanent record of each spectral map, meaning that data can be re-interrogated for new peptides of interest *in silico* as interactions become clearer following analysis.

There have been previous reports of protein biomarkers obtained using large-scale shotgun proteomics techniques associated with treatment response in RA [7], however, these studies have been limited by heterogeneous treatment populations (i.e. patients were on more than one drug, but grouped together for analysis), small sample sizes and no comparisons to healthy controls (HCs). Proteins such as monocyte chemoattractant protein (MCP)-1 [8], epidermal growth factor (EGF) [8], vitamin K–dependent protein S (PROS) [9] and E3 ubiquitin-protein ligase carboxyl terminus of heat shock cognate 70-interacting protein (CHIP) [9] were identified, but these were small-scale studies with <35 patients included. Furthermore, none of these proteins have been consistently replicated in independent prospective validation cohorts with a large sample size.

The aim of the current study was to identify protein biomarkers predictive of treatment response to the bDMARD etanercept in patients with RA and to use network-based methods to identify relevant pathways.

## Methods

### Patient and public involvement

Prior to implementation, the study concept and design were discussed and developed in conjunction with the Research User Network (comprising patients with various rheumatological conditions, including RA), based within the Centre for Musculoskeletal Research (CfMR) at the University of Manchester.

### Study participants

Patients with RA according to the 1987 ACR classification criteria were recruited to the Biologics in Rheumatoid Arthritis Genetics and Genomics Study Syndicate [BRAGGSS; Research Ethics Committee (REC) reference 04/Q1403/37], a prospective multicentre observational study based in the UK [10]. This study was in compliance with the Declaration of Helsinki and all participants gave written informed consent. The study cohort consisted of participants recruited to the prospective arm of BRAGGSS who were commencing etanercept or an etanercept biosimilar. Participants were Caucasian, bDMARD naïve, had a pretreatment 28-joint DAS (DAS28) >5.1 (indicative of high disease activity) and were ≥18 years of age. In order to be eligible for analysis, each participant required serum to be available at two time points, pretreatment (baseline) and following 3 months on the drug, in addition to RA clinical disease outcome measures available at baseline and 3 and 6 months. Participants were recruited between 2009 and 2016 from secondary care rheumatology departments in the 60 centres participating in BRAGGSS, with follow-up concluding 12 months after initial recruitment into the study for each participant. Participants were opportunistically recruited over several years and a sample size calculation was not applied.

In all RA participants, clinical data and DAS28 were available. DAS28 was calculated using a four-component algorithm consisting of tender joint count (TJC) and swollen joint count (SJC) of 28 joints, patient visual analogue scale of global health (VAS-GH, 0–100 mm) and high-sensitivity CRP (hsCRP) measured using ELISA at the National Institute for Health and Care Research (NIHR) National Biosample Centre (Milton Keynes, UK).

HCs were recruited from the National Repository Study (REC reference 99/8/084), a study consisting of healthy volunteers to provide samples for comparison cohorts and protocol, technique and method development.

### Sample processing

All patient serum samples were processed at the CfMR and by CfMR laboratory staff. Blood tubes were spun at 1720 *g* for 10 min, then serum was extracted into aliquots before being frozen at −80°C. RA participant samples were received either on the same day (from centres close to the CfMR geographically) or mostly through the UK postal service (Royal Mail), with a median time between collection and sample processing of 2 days (interquartile range 1–3). All HC samples were collected onsite and processed on the same day at the CfMR.

Frozen serum aliquots were transferred to the Stoller Biomarker Discovery Centre (SBDC, Manchester, UK), where protein spectral maps were extracted for each sample using SWATH-MS, using techniques for sample processing and data acquisition as previously described [11, 12]. Samples were processed by the SBDC; SWATH-MS acquisition is discussed in more detail in the Supplementary Methods, available at *Rheumatology* online.

### Statistical analysis

All analyses were carried out in R version 4.0.2 (R Foundation for Statistical Computing, Vienna, Austria) [13]. Proteomics data were pre-processed, including imputation of missing protein values, as described in the Supplementary Methods, available at *Rheumatology* online. Peptide spectra were identified from samples using both a generic open-access plasma library, as well as a bespoke library of proteins curated from pre-existing literature on proteomics studies in patients with RA.

#### Differentially expressed proteins between cases and controls

Differential expression of proteins between RA patients (cases) at baseline and HCs was calculated using a Welch’s *t*-test implemented by using the col_t_welch function in the MatrixTests [14] package. In order to reduce the high dimensionality of the dataset, only proteins that were statistically differentially expressed (*P* < 0.05) between patients with active RA and HCs, who represented a healthy physiological state, were retained, meaning that these proteins were significantly increased or decreased in cases compared with controls. Therefore, proteins for investigation were selected on the basis that they showed differential expression in samples from RA patients compared with HCs.

#### Association of protein expression with clinical outcome measures

Primary analysis was carried out in a discovery cohort of patients with RA. The R base package was used to carry out regression between expression of each protein and the following continuous RA disease outcomes:

- Primary outcome measures: EULAR response criteria [15] (poor *vs* good/moderate) and DAS28 remission (i.e. DAS28 <2.6) [16]—logistic regression.
- Secondary outcome measures: DAS28 and its subcomponents (TJC, SJC, VAS-GH, hsCRP)—linear regression.

In linear regression analysis, a positive β-coefficient indicated a positive association between a protein and the clinical outcome measure of interest and a negative coefficient indicated an inverse association, i.e. as a clinical outcome measure value increased, the protein value decreased. Both univariate and multivariable analyses were carried out for each protein. The following variables were included as potential confounding covariates: age at baseline, RA disease duration prior to starting etanercept, biological sex, concurrent csDMARD therapy, BMI, seropositivity of either RF or ACPA, pretreatment (baseline) DAS28, systemic corticosteroid use within ±12 weeks of starting etanercept (intramuscular and oral administration) and the presence of the following comorbidities: cardiovascular disease, respiratory disease, liver disease, renal disease, diabetes and malignancy; these are expanded on further in the Supplementary Methods, available at *Rheumatology* online. Adjustment for false discovery rate due to multiple testing was carried out using the Benjamini–Hochberg procedure [17]. Significantly associated proteins (following multiple testing adjustment with *P* < 0.05) were then added into a multivariable model adjusting for the same confounding covariates. Proteins at baseline (pretreatment) were compared with outcomes at 3 and 6 months and proteins at 3 months were compared with outcomes at 6 months.

For validation of the most relevant findings from the primary analysis, peptide spectra for prioritized proteins were extracted from SWATH-MS spectral maps generated in an independent cohort of patients with RA. Statistical analysis was repeated as detailed above. Technical validation with an orthogonal method was carried out using a Pearson correlation between log2-transformed hsCRP (measured by ELISA at the NIHR National Biosample Centre) against CRP measured using SWATH-MS in the main discovery cohort of patients.

### Subnetwork analysis

Subnetwork analysis was carried out on all proteins from the regression analysis that remained significant following adjustment in multivariable models that included potential confounders. First, enrichment analysis was carried out using Enrichr [18], then potential interactions with these significant proteins were determined using the Disease Module Detection (DIAMOnD) algorithm [19]. The optimal parameters for the subnetwork construction were determined using a grid search over the number of subnetwork proteins and the α-value (weighting applied to seed proteins), where the parameters giving the lowest biological validation *P*-value were used to generate the overall subnetwork. Biological validation refers to validating the generated subnetwork against the list of significant proteins identified following enrichment analysis. Detailed methods are outlined in the Supplementary Methods, available at *Rheumatology* online.

## Results

### Study participants

Samples from 180 patients with RA were included in the discovery cohort and from 58 patients in the validation cohort; their summary characteristics are detailed in Table 1. Patients who were not on concurrent csDMARDs, as detailed in Table 1, had been commenced on etanercept monotherapy after failing conventional csDMARD escalation, as per National Institute for Health and Care Excellence guidance [20].

**Table 1.** Baseline characteristics of patients recruited to the study

Characteristic	Value
Discovery cohort (*n* = 180)	
Female, *n* (%)	134 (74.44)
Age, years, median (IQR)	56.90 (49.96–64.93)
Disease duration prior to starting bDMARD, years, median (IQR)	6 (2–14)
Body mass index, kg/m^2^, median (IQR)	27.56 (23.86–32.54)
Concurrent csDMARD, *n* (%)	147 (81.67)
DAS28, median (IQR)	5.9 (5.3–6.4)
Ever seropositive (RF and/or ACPA), *n* (%)	120 (66.67)
Validation cohort (*n* = 58)	
Female, *n* (%)	44 (75.86)
Age, years, median (IQR)	58.18 (51.65–66.43)
Disease duration prior to starting bDMARD, years, median (IQR)	6 (3–10)
Body mass index, kg/m^2^, median (IQR)	28.38 (23.51–35.06)
Concurrent csDMARD, *n* (%)	52 (89.66)
DAS28, median (IQR)	5.97 (5.49–6.77)
Ever seropositive (RF and/or ACPA), *n* (%)	39 (67.24)

IQR: interquartile range.

### Differential expression of proteins between RA cases and HCs

Pretreatment samples reflecting high RA disease activity from the 180 RA patients were compared with HCs (*n* = 14) and 216 of 482 proteins were found to be significantly differentially expressed between the two groups. A total of 70 of the 216 proteins were down-regulated in RA patients with active disease compared with HCs and the remaining proteins were all up-regulated. The full results are presented in Supplementary Table S1 (available at *Rheumatology* online). These 216 proteins were then prioritized in subsequent analyses.

### Logistic regression models of protein expression associated with EULAR response and DAS28 remission (primary outcome measures)

Following adjustment in multivariable models, no proteins were associated with EULAR response (Table 2). T-complex protein 1 subunit η (TCPH; UniProt identifier Q99832) at baseline was found to be associated with reduced odds of achieving DAS28 remission at 3 months [adjusted odds ratio (OR_adj_) 0.32 (95% CI 0.11, 0.85), adjusted *P* (*P*_adj_) = 2.91E-02]. The full results of confounder-adjusted and multivariable analyses are presented in Supplementary Tables S2 and S3 (available at *Rheumatology* online). Figures demonstrating whether models met the assumptions of regression are presented in Supplementary Figs S1–S3 (available at *Rheumatology* online).

**Table 2.** Proteins associated with RA clinical outcome measures after treatment with etanercept, adjusted in multivariable models

DAS28 remission (<2.6)				
Protein	Protein measurement time point	Outcome measure time point	OR_adj_ (95% CI)	Adjusted *P*-value
TCPH (Q99832)	Baseline	3 months	0.32 (0.11, 0.85)	2.91E-02

Protein	Protein measurement time point	Outcome measure time point	β-coefficient_adj_ (95% CI)	Adjusted *P*-value

DAS28				
EHD1 (Q9H4M9)	Baseline	3 months	0.21 (0.05, 0.37)	9.49E-03
TCPH (Q99832)	Baseline	3 months	0.62 (0.16, 1.08)	9.59E-03
CRP (P02741)	3 months	6 months	0.16 (0.01, 0.30)	3.93E-02
C9 (P02748)	3 months	6 months	0.38 (0.01, 0.76)	4.74E-02
hsCRP measured using ELISA				
SELENOP (P49908)	Baseline	6 months	−5.68 (−9.19, −2.17)	1.87E-03
MAP2K3 (P46734)	Baseline	6 months	7.85 (3.49, 12.22)	5.79E-03
	3 months	6 months	6.30 (1.72, 10.89)	7.93E-03
CLTC (Q00610)	Baseline	6 months	4.76 (0.77, 8.75)	2.08E-02
SAA1 (P0DJI8)	3 months	6 months	3.24 (1.22, 5.26)	2.00E-03
MYLK (Q15746)	3 months	6 months	8.92 (1.13, 16.72)	2.64E-02
VAS-GH				
ASPH (Q12797)	3 months	6 months	−0.01 (−0.02, −0.01)	3.14E-02

UniProt identifiers are included in parentheses after each protein abbreviation.

CLTC: clathrin heavy chain 1; C9: complement component C9; EHD1: EH domain-containing protein 1; MCID: minimally clinically important difference; MYLK: myosin light chain kinase; SELENOP: smooth muscle; selenoprotein P; SAA1: serum amyloid A-1 protein.

### Linear regression models of protein expression associated with RA disease outcome measures (secondary outcome measures)

Following adjustment of linear outcome measures in multivariable models, a number of proteins were found to be associated with clinical outcome measures (Table 2):

- Four proteins were associated with DAS28.
- Five proteins were associated with hsCRP measured using ELISA. Dual specificity mitogen-activated protein kinase 3 (MAP2K3) at both baseline and 3 months was associated with hsCRP at 6 months.
- Aspartyl/asparaginyl β-hydroxylase (ASPH; UniProt identifier Q12797) was associated with VAS-GH.

The full results of confounder-adjusted and multivariable analyses are presented in Supplementary Tables S4–S12 (available at *Rheumatology* online). Figures demonstrating whether models met the assumptions of regression are presented in Supplementary Figs S4–S12 (available at *Rheumatology* online).

### Validation in an independent cohort (SWATH-MS acquisition)

Validation of significant proteins identified from the previous section was carried out in an independent cohort using the original multivariable models from the discovery cohort. TCPH measured at baseline remained significantly associated with reduced odds of DAS28 remission at 3 months [OR_adj_ 0.06 (95% CI 0.00, 0.50), *P*_adj_ = 2.71E-02]. MAP2K3 measured after 3 months of treatment also remained significantly associated with hsCRP measured using ELISA after 6 months of treatment [β-coefficient_adj_ 9.39 (95% CI 0.44, 18.33), *P*_adj_ = 4.83E-02]. The remaining proteins did not replicate in this smaller cohort. Full results are available in Supplementary Table 13 (available at *Rheumatology* online).

### Technical validation of CRP measured by SWATH-MS using an orthogonal method

At baseline, CRP measured using SWATH-MS was significantly correlated with log2-transformed CRP measured using ELISA [Pearson’s correlation coefficient 0.88 (95% CI 0.83, 0.91), *P* ≤ 2.2E-16]. This was also the case after 3 months of treatment [Pearson’s correlation coefficient 0.80 (95% CI 0.73, 0.86), *P* ≤ 2.2E-16]. See Supplementary Figs S13 and S14 (available at *Rheumatology* online) for scatter plots of these data. This demonstrates close agreement between protein quantification using both SWATH-MS and ELISA.

### Subnetwork analysis

**Figure 1.**
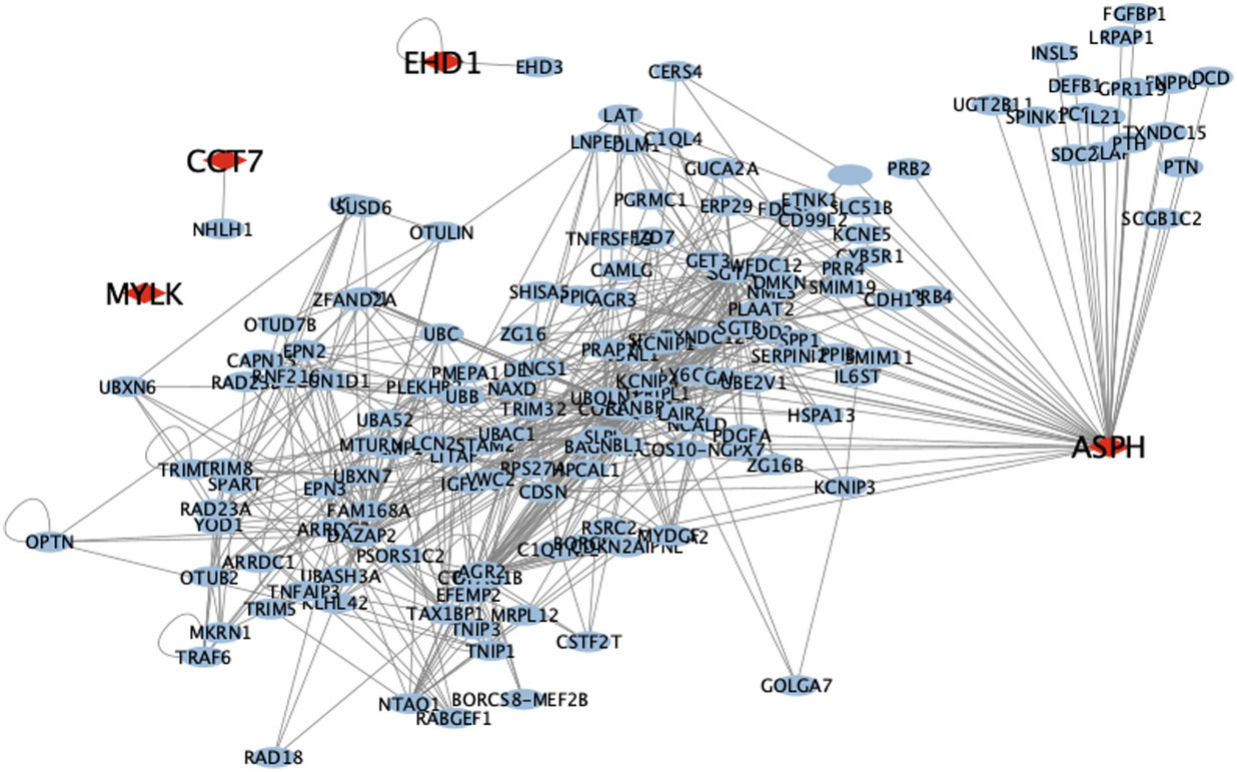
Diagrammatic representation of selected protein subnetwork based on the 10 significant proteins from regression analysis. The diamonds represent significant proteins from the regression analysis that are also present in the Human Interactome, which have been used as input nodes for the sub-network analysis. The ovals represent additional proteins identified from sub-network analysis using the Human Interactome

**Figure 2.**
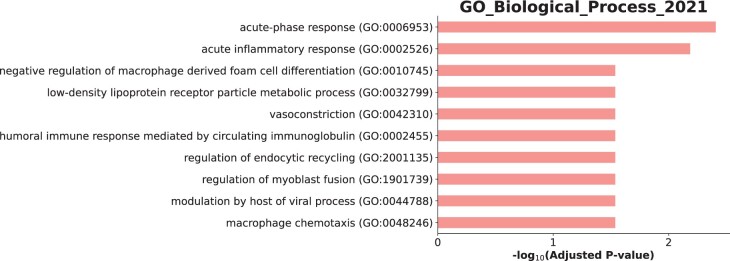
Summary of the ontological themes of the 157 subnetwork genes following enrichment analysis

Four of the ten proteins correlated with RA clinical disease outcomes were present in the Human Interactome [21], to which the DIAMOnD algorithm was applied. The most parsimonious network giving the lowest biological validation *P*-value was chosen, consisting of 157 genes and α = 3 (Fig. 1). Aspartyl/asparaginyl β-hydroxylase (ASPH; one of the seed nodes) was found to be a hub/influential node within the subnetwork. Fig. 2 summarizes the ontological themes of the 157 genes following enrichment analysis, with the strongest associations being with acute-phase response and acute inflammatory response.

## Discussion

In the largest study of proteomic biomarkers compared with clinical outcome measures in patients with RA treated with etanercept to date, we report that four protein markers measured pretreatment associate with one or more measures of outcome by 3 or 6 months and six protein markers measured after 3 months of treatment associate with one or more measures of outcome by 6 months. Subnetwork analysis found 157 genes associated with the proteins identified, with enrichment analysis giving acute-phase response and acute inflammatory response as the most significantly associated pathways.

TCPH is a protein of note in this study, as its measurement at baseline was associated with DAS28 remission after 3 months of treatment, and this finding was replicated in an independent cohort. TCPH is involved in processes during adenosine triphosphate (ATP) hydrolysis [22, 23]. Pretreatment levels of TCPH were also associated with DAS28 by 3 months. ATP hydrolysis is the process by which energy is released in the conversion of ATP to adenosine diphosphate. Given that active inflammation is a high-energy state, this putative pretreatment biomarker may simply reflect the level of pretreatment inflammation, which is known to correlate with response to treatment, i.e. a higher pretreatment DAS28 correlates with a greater improvement due to regression to the mean. However, TCPH [β-coefficient 0.01 (95% CI 0.00, 0.01), *P*_adj_ = 0.28] was not correlated with pretreatment CRP levels, and further research will be required to determine whether the association of TCPH with clinical outcome measures is specific to etanercept response in RA or is a general theragnostic indicator.

MAP2K3 is also an interesting protein in this study, as its measurement at both baseline and 3 months was associated with future hsCRP at 6 months. MAP2K3 is a dual-specificity kinase that is activated via cytokines and environmental stress [24]. Its association with future hsCRP could implicate it as a potential biomarker of systemic inflammation despite treatment with etanercept; it could be hypothesized that patients with increased levels may be prone to uncontrolled inflammation (measured by hsCRP as a proxy) despite treatment. Its lack of association with EULAR response or DAS28 could be because of inclusion of DAS28 subcomponents (TJC, VAS-GH) [25] that do not reflect systemic inflammation as well as hsCRP.

Findings from this study have not replicated previously identified proteins that were reported to show an association with treatment response to etanercept in patients with RA [8, 9]. This failure to replicate previous findings of other proteomics studies may be due to a number of reasons, such as heterogeneous populations of study (e.g. patients with different disease duration, disease severity, therapy prior to commencing bDMARD therapy, ethnicity etc.), as well as varying methods of proteomics acquisition. There are advantages and disadvantages to the use of various proteomics acquisition techniques, and some higher-throughput methods may not capture the full proteome during sample processing [26].

Findings from the subnetwork analysis showed that ASPH was an influential node in the overall subnetwork. It is interesting that this protein was associated with patient global health, indicating that there might be a biological component underlying this patient-reported outcome measure. ASPH (UniProt identifier Q12797) is a protein with two known isoforms: isoform 1 is involved in hydroxylation of Asp/Asn residues in specific epidermal growth factor–like domains [27] and isoform 8 is a membrane-bound calcium ion–sensing protein that is part of the endoplasmic reticulum (ER) [28]. The ER is the major protein synthesis site of the cell and disruption of normal ER homeostasis leads to a condition of physiological stress called ER stress [29]. ER stress precipitates an intracellular process termed unfolded protein response (UPR), which has the aim of re-establishing ER homeostasis and can result in either cell survival or death. Triggers for UPR activation include hypoxia, hypoglycaemia and genome instability, which are all physiological conditions that can be present during an active systemic inflammatory response [30–32], such as that of active RA. Subnetwork analysis also identified acute-phase response and acute inflammatory response as the most significant pathways implicated by the protein subnetwork, which would agree with current knowledge on RA pathophysiology [34] and would explain the significant associations between five proteins and future hsCRP measurements.

This study has a number of strengths, including the fact that it is a large, prospectively recruited cohort on a single bDMARD and analysis included 216 proteins shown to be differentially expressed between RA patients and HCs. SWATH-MS is a stable and reproducible method of proteomics acquisition, as it relies on destructive enzymatic digestion of proteins prior to MS and it is not limited by pre-selection of proteins of interest in the same way as proprietary multiplexed panels. The agreement with hsCRP values acquired using ELISA has been demonstrated from our data. A further strength of this study is the replication of associations of the proteins TCPH and MAP2K3 in an independent cohort.

However, there are a number of limitations. Multiple comparisons were performed, making the chance of false-positive findings higher, but significance thresholds were adjusted using the Benjamini–Hochberg correction in order to mitigate this. Only the 216 differentially expressed proteins were included in the analysis, but there may be proteins that are not significantly differentially expressed that correlate with treatment response. Given that SWATH-MS provides a permanent spectral map of all theoretical proteins in a biological sample, further proteins could be selected for testing in the future, based on previous or emerging reports of an association with treatment response, e.g. following subnetwork analysis. Finally, there was delay in sample processing for the majority of RA postal samples, but not HC samples. However, analysis of the delay to processing (data not presented) showed that inclusion of delay as a confounding variable did not affect the results. This may demonstrate utility in our findings, as they may be more likely to translate into the National Health Service (NHS), as our study protocol reflects delays in processing within the health service. Future replication of findings in an independent prospective cohort may lead to translation of predictive biomarkers of treatment response to etanercept into clinical practice.

In conclusion, in this longitudinal study of patients with RA we have identified candidate protein biomarkers of treatment response to etanercept measured using SWATH-MS, two of which were replicated in an independent dataset. Further validation and assessment of predictive utility will be required before translation into clinical practice.

## Supplementary Material

kead321_Supplementary_Data

## Data Availability

Data are available upon request to the corresponding author.
