# Supplementary material for: A proteomics study of rheumatoid arthritis patients on etanercept identifies putative biomarkers associated with clinical outcome measures
Source: Rheumatology (Oxford). 2023 Jun 30;63(4):1015–21. doi: 10.1093/rheumatology/kead321 (PMC10986807; doi:10.1093/rheumatology/kead321)
Supplement: kead321_Supplementary_Data [file kead321_supplementary_data.docx]

**SUPPLEMENTARY MATERIAL**

**Supplementary methods**

*SWATH-MS sample preparation*

Sample preparation and SWATH-MS for discovery proteomics was carried out at the Stoller Biomarker Discovery Centre (SBDC), The University of Manchester, UK. A dataset of extant samples from patients treated with etanercept/etanercept biosimilars from the wider BRAGGSS cohort was generated by SBDC as well as samples from 14 healthy control (HC) participants; this was processed in three batches.

Samples were transferred from CfMR to SBDC on dry ice. Following thorough thawing, serum samples were depleted of abundant proteins (e.g. albumin, IgA, IgG, IgM). Within each batch, all samples were plated in a random order and control samples were used at the beginning, during and at the end of MS runs in order to detect run-order effects. The third batch of samples were depleted of the top 14 most abundant proteins using commercially-available HighSelect™ Top14 Abundant Protein Depletion Mini Spin Columns (Thermo Fisher Scientific, Waltham, MA, USA), according to the manufacturer’s methods. The remaining BRAGGSS etanercept samples were depleted of the top 12 most abundant proteins using commercially-available Top-12 kits (Pierce, Thermo, Loughborough, UK), according to the manufacturer’s methods. Different immunodepletion kits were used as the SBDC protocol had been updated before the later batch of samples was processed.

Protein amount was then assayed in the resultant solution using a Bradford reagent (Bio-Rad, Watford, UK). Solution containing 40μg of protein was subsequently processed further. The third batch of samples were reduced, alkylated and digested by using S-trap columns prior to lyophilisation. For the remaining samples, samples were reduced using 60 mM tris (2-carboxyethyl) phosphine at 60oC for 60 minutes, and then were alkylated using 10 mM iodoacetamide for 30 minutes in the dark. Protein digestion was carried out overnight using trypsin (Promega, Southampton, UK) at 37oC in a 10:1 ratio of protein-to-enzyme.

For all samples, once digested, peptides were cleaned using a SepPak (Waters, Wilmslow, UK) 96-well plate solid-phase extraction system. Following digestion, gel electrophoresis was then carried out in order to assess the efficiency of immunodepletion and digestion, and to identify any discrepancies or handling errors during prior sample processing. Each gel was run using a Mini Gel Tank containing Bolt 4 – 12% Bis-Tris gel and SDS Running Buffer (all Thermo Fisher Scientific, Waltham, MA, USA). Tanks were supplied with a PowerEase® 90W Power Supply (also Thermo Fisher). Gels were assessed for adequacy of digestion: digested proteins should be small enough to run to the end of the gel.

*Bespoke RA protein library generation*

A library of proteins associated with RA as determined from previous studies was generated following a detailed literature search and review by the author. The Ovid MEDLINE database was searched from 1946 until October week 5 2018 using the following search terms:

- “Rheumatoid arthritis” as a keyword; “Arthritis, Rheumatoid” as a subject heading (all subheadings included) – search **1**.
- “Proteomics” as a keyword; “Proteomics” as a subject heading (all subheadings included) – search **2**.
- “Proteins” as a keyword (all subheadings included) – search **3**.
- Combine searches **2** OR **3** – search **4**.
- Combine searches **1** AND **4** – search **5**.

Studies were included if they were carried out in human participants with RA, with at least 10 participants in each comparison group. The full text of each manuscript had to be available via The University of Manchester Library. In addition, only proteins that were specifically identified were included; spectral peaks alone were excluded. Review articles, studies utilising only cell lines, clinical trials and studies of DNA and RNA were also excluded. A library of proteins from the remaining included studies was compiled, and identifier (ID) numbers were allocated following a search of the Universal Protein Resource (UniProt)^1^ database.

In addition to proteins identified from the above literature search, proteins involved in the TNF pathway according to the Kyoto Encyclopedia of Genes and Genomes (KEGG)^2^ were included.

*SWATH-MS acquisition*

Samples were analysed by SWATH-MS with a micro-flow LC-MS system, comprising an Eksigent nanoLC 400 autosampler and an Eksigent nanoLC 425 pump, coupled to a SCIEX 6600 TripleTOF mass spectrometer (all equipment listed: SCIEX, Framingham, MA, USA). The LC method consisted of a 120-minute gradient between a buffer A of 98% water, 2% (volume per volume, *v/v*) acetonitrile and 0.1% (weight per volume, *w/v*) formic acid, and a buffer B of 80% acetonitrile, 20% water and 0.1% formic acid. Samples were injected in duplicate. Spectra were acquired with the mass spectrometer in SWATH mode, using the 100 variable window method, with MS2 windows ranging from 399.5 to 1,249.5 *m/z*, with optimised collision energy equations. The MS1 mass range was from 100 to 1,500 *m/z*, with an accumulation time of 0.05 seconds and a cycle time of 2.6 seconds.

Spectral data files were then converted using wiffconverter (SCIEX, Framingham, MA, USA) to mzML format, an open extensible markup language (XML) format that has been collaboratively developed specifically for use in MS data output files^3^. A protein library search was then carried out using OpenSWATH v.2.0.0^4^ against both a publically available twin plasma library^5^ (version published 5^th^ January 2015) and a bespoke library of proteins associated with RA (as detailed in Section 3.2.3). OpenSWATH results files were then processed using PyProphet^6^, an algorithm developed for targeted proteomics, particularly large-scale data generated from OpenSWATH or DIANA^6^ (an alternative set of bioinformatics tools for analysis of SWATH data). Following processing with PyProphet, files were then aligned using the feature alignment script from the MS Proteomics Tools^7^ online repository of bioinformatics tools developed to aid analysis of proteomic MS data. A target false discovery rate (FDR) was set at 0.01 at the peptide-spectrum match (PSM) level.

Further data pre-processing was carried out using R v.3.4.1, using Bioconductor v.3.5^8^ packages MSstats^9^ and SWATH2Stats^10^ for downstream processing. Coefficients of variation (CV) were calculated between technical replicates; any samples with a median CV of ≥ 20% were re-run. Data were filtered by m-score (also known as q-value; this constitutes the minimum FDR acceptable in samples). m-scores were generated using the filter_mscore_fdr function in the SWATH2stats package; the overall protein FDR target was set at 0.02 and the upper overall peptide FDR limit was set to 0.05. Data were then converted from a feature alignment input to MSstats input using the convert4MSstats function in SWATH2stats. MSstats was then used to normalise and summarise protein intensity data, using the dataprocess function with default arguments. Protein intensity readings were then log2-transformed and centred and scaled, so that all coefficients were scaled within each batch that was processed.

*Quality control of SWATH-MS data*

Quality control (QC) of spectral maps generated for each sample was carried out, as samples were processed in three batches. QC of proteomics data was carried out in R v.4.0.2, primarily in the dataset generated from second batch of samples to be processed by SBDC, as this batch included the HC samples. Firstly, data readouts from both plasma and bespoke RA libraries were merged for each study participant and duplicate proteins removed, using the tidyverse^11^ package. Proteins with near-zero variance across all participants were removed using the caret^12^ package. Density plots were generated in order to visually compare mean expression of proteins versus levels of protein missingness i.e. <25% missing, 25-50% missing, 50-75% missing, >75% missing. Heatmaps were generated using the ComplexHeatmap^13^ package to visually inspect for any patterns of protein missingness e.g. by time point or treatment response.

Once it had been ascertained that missing proteins were likely missing at random (for example, due to not being captured in a particular SWATH window at a given time point), missing proteins were imputed. Prior to imputation, accuracy of various imputation methods was carried out on a subset of complete data with no missing values. The simIm function of the imputeR^14^ package was used to randomly spike 30% missing values into the data subset, before several different methods of imputation were compared for accuracy:

• Lasso regression, a form of linear regression where data values are shrunk towards a central point, such as the mean. This method favours more sparse models with fewer parameters. The lassoR function was used in the imputeR package.

• Partial least squares regression, another form of linear regression that is similar to principal components analysis (PCA), where predicted and observed variables are projected into a new theoretical space. The plsR function was used in the imputeR package.

• K-nearest neighbours, a machine learning algorithm that assumes similarity of data points in close proximity. The knn.impute function was used in the bnstruct^15^ package.

• Multiple imputation by chained equations (MICE), a multiple imputation method that assumes that data is missing at random, with prediction of new data based on a regression model. This was carried out using the mice^16^ package.

• Random forest, an ensemble machine learning algorithm that utilises multiple decision trees. This was carried out using the MissForest^17^ package.

The root mean square error (RMSE) was used to determine the most accurate method of imputation; the lower the value, the better the accuracy. Using this metric, random forest was selected as the imputation method of choice.

The above random forest algorithm was then used to impute missing values in the wider dataset. This was carried out separately in each batch, at each time point. It was hypothesised that levels of any given protein would change over time, so imputation was carried out separately at each time point in order to increase imputation accuracy; imputed values would not then be affected by increased or decreased expression of the same protein at different time points. Following imputation, further density plots were created for data pre- and post-imputation to visually inspect whether imputation altered protein expression densities. Hierarchical clustering was used to identify and remove any outlying samples. Then, using the caret package, PCA was carried out to determine whether there was any batch effect in protein expression. Finally, the ComBat function in the sva^18^ package was used in order to correct for batch effect in the entire imputed dataset.

*Selection of potential confounding variables as covariates*

The following table outlines which potential confounders were selected for inclusion in adjusted models, and the justification for the inclusion of each:

| **Potential confounding variable** | **Justification for inclusion** |
| --- | --- |
| Age at baseline | Age has been shown to be associated with increased disease activity^19^. |
| RA disease duration prior to starting etanercept | Disease duration prior to starting tumour necrosis factor inhibitors (TNFi) has previously been shown to be associated with decreased treatment response^20^. |
| Biological sex | Female patients have been shown to be less likely to achieve remission^21^. |
| Concurrent conventional synthetic disease-modifying anti-rheumatic drug (csDMARD) therapy | Current European Alliance of Associations for Rheumatology (EULAR) consensus guidance is that biologic DMARDs (bDMARDs) should be given in combination with a csDMARD as bDMARDs have been shown to have superior efficacy when given in combination with a csDMARD, compared with monotherapy^22^. |
| Body mass index (BMI) | Raised BMI has been shown to be associated with reduced rates of RA remission, regardless of therapeutic agent^23^. |
| Seropositivity of either rheumatoid factor or anti-citrullinated peptide antibodies (ACPA) | Both rheumatoid factor and ACPA seropositivity have been identified as poor prognostic factors from RA trial data^24^. |
| Pre-treatment Disease Activity Score of 28 Joints (DAS28) | A statistical adjustment for initial DAS28 is required due to the use of DAS28 to calculate primary outcome measures after treatment. |
| Systemic corticosteroid use within ± 12 weeks of starting etanercept | Patient receiving concurrent systemic corticosteroids are likely to have increased disease activity and potentially a higher systemic inflammatory burden^22^. |
| Comorbidities:   - Cardiovascular disease: hypertension, angina, previous myocardial infarction, stroke only. - Respiratory disease: asthma, chronic obstructive pulmonary disease (COPD), tuberculosis (TB) only. - Any liver disease. - Any renal disease. - Diabetes (type 1 and type 2). - Previous or current malignancy (solid tumours and haematological). | Comorbidities in patients with RA are associated with poor health outcomes and increased mortality, although the physiologically mechanism is unclear^25^. |

*Sub-network analysis*

Significant proteins from the regression analyses were used as input for the DIAMOnD algorithm^26^ to identify a sub-network within the Human Interactome^27^, a reference interactome map of human binary protein interactions. Significant proteins found in the Human Interactome (n = 5) were used as the seed nodes in DIAMOnD. The optimal parameters for the sub-network generation were determined using a grid search over the number of sub-network proteins and the α value, where the parameters giving the lowest biological validation p-value were used to generate the overall sub-network. Biological validation refers to validating the generated sub-network against the list of significant proteins. This was done as follows:

1. Performance of enrichment analysis using Enrichr^28^ using the list of significant proteins which serve as seed nodes for DIAMOnD. A list of enriched GO terms for biological processes was obtained.
2. From the above GO terms, all relevant proteins were extracted and acted as the enriched protein set.
3. During the grid search process, a new set of sub-network proteins was generated. Fisher’s exact test was performed on this set of sub-network proteins, as well as the enriched protein set, with the list of Human Interactome proteins as a reference. This was done to statistically identify the significant of associations between sub-network proteins and enriched proteins, with the aim of selecting a list of proteins that would be closely linked to the seed nodes. For each iteration of the grid search, the same number of proteins were used for the Fisher’s exact test. As there were only 5 proteins used in the initial enrichment analysis, we used the protein in the interval of [*i* – 5/2, *i* + 5/2], where *i* is the number of sub-network proteins being generated, and the protein list here is sorted by order of generation by the DIAMOnD algorithm. This was done to account for the dependence of p-values on the set size being tested.

**Supplementary Results**

*Supplementary Table S1. Significantly differentially expressed proteins in patients with RA compared to HC.*

| **Protein name (UniProt ID)** | **Mean expression RA** | **Mean expression HC** | **Difference in means (95% CI)** | **Standard error** | **p-value** | **% missing before imputation** |
| --- | --- | --- | --- | --- | --- | --- |
| PARK7 (Q99497) | 13.50 | 7.06 | 6.44 (6.41-6.47) | 1.40E-02 | 2.83E-111 | 72.05 |
| ENO1 (P06733) | 11.28 | 2.15 | 9.13 (9.08-9.19) | 2.62E-02 | 9.28E-104 | 64.10 |
| RTCB (Q9Y3I0) | 12.39 | 5.00 | 7.39 (7.35-7.43) | 2.18E-02 | 4.93E-103 | 67.18 |
| XRCC6 (P12956) | 12.44 | 4.90 | 7.54 (7.49-7.58) | 2.30E-02 | 3.99E-102 | 60.26 |
| KCC1A1 (Q14012) | 13.99 | 6.23 | 7.76 (7.71-7.81) | 2.41E-02 | 1.31E-101 | 62.82 |
| CALD1 (Q05682) | 11.96 | 4.39 | 7.57 (7.52-7.62) | 2.37E-02 | 1.84E-101 | 76.67 |
| PPIA (P62937) | 13.07 | 4.52 | 8.55 (8.49-8.60) | 2.69E-02 | 2.56E-101 | 60.77 |
| EHD1 (Q9H4M9) | 12.83 | 5.15 | 7.68 (7.63-7.73) | 2.45E-02 | 6.33E-101 | 72.82 |
| S100A8 (P05109) | 15.46 | 3.40 | 12.05 (11.95-12.16) | 5.19E-02 | 6.90E-93 | 34.36 |
| RSU1 (Q15404) | 11.81 | 5.41 | 6.40 (6.34-6.47) | 3.35E-02 | 1.37E-87 | 58.21 |
| NAMPT (P43490) | 12.70 | 3.91 | 8.80 (8.66-8.93) | 6.80E-02 | 3.94E-77 | 53.08 |
| TFR1 (P02786) | 12.87 | 4.14 | 8.72 (8.58-8.87) | 7.30E-02 | 5.39E-75 | 39.23 |
| ELNE (P08246) | 11.17 | 4.82 | 6.34 (6.21-6.47) | 6.52E-02 | 1.58E-69 | 62.31 |
| NNRE (Q8NCW5) | 10.49 | 3.78 | 6.71 (6.50-6.91) | 1.03E-01 | 7.90E-59 | 65.13 |
| BLVRB (P30043) | 10.56 | 4.77 | 5.77 (5.58-5.96) | 9.45E-02 | 4.22E-57 | 77.95 |
| PPIB (P23284) | 10.14 | 3.97 | 6.17 (5.89-6.46) | 1.43E-01 | 7.22E-48 | 52.31 |
| MIME (P20774) | 8.75 | 9.72 | -0.97 (-1.03-(-0.91)) | 2.92E-02 | 2.74E-41 | 77.69 |
| B3AT (P02730) | 10.60 | 8.85 | 1.75 (1.60-1.89) | 7.17E-02 | 1.94E-33 | 82.05 |
| S100A6 (P06703) | 10.21 | 8.94 | 1.26 (1.17-1.36) | 4.87E-02 | 2.66E-29 | 68.72 |
| GTF2I (P78347) | 13.03 | 11.48 | 1.55 (1.39-1.71) | 8.02E-02 | 3.92E-28 | 70.51 |
| S100A9 (P06702) | 11.83 | 9.45 | 2.39 (2.12-2.65) | 1.32E-01 | 2.48E-26 | 43.08 |
| HSP90A (P07900) | 15.63 | 14.48 | 1.14 (1.00-1.29) | 8.03E-02 | 3.23E-26 | 66.67 |
| LRRFIP1 (Q32MZ4) | 14.81 | 13.88 | 0.92 (0.81-1.04) | 5.99E-02 | 6.68E-25 | 72.56 |
| PDIA6 (Q15084) | 13.04 | 11.51 | 1.52 (1.33-1.72) | 9.74E-02 | 3.34E-23 | 58.46 |
| BGAT1 (O43505) | 17.12 | 15.77 | 1.35 (1.16-1.53) | 9.25E-02 | 1.09E-21 | 70.26 |
| COL6A2 (P12110) | 13.36 | 14.72 | -1.36 (-1.56-(-1.16)) | 1.00E-01 | 1.40E-21 | 60.77 |
| FCGBP (Q9Y6R7) | 12.41 | 11.20 | 1.21 (1.04-1.39) | 8.70E-02 | 3.14E-21 | 77.69 |
| TPM4 (P67936) | 10.70 | 8.64 | 2.06 (1.76-2.36) | 1.50E-01 | 8.54E-21 | 28.97 |
| TPM3 (P06753) | 15.15 | 14.10 | 1.05 (0.86-1.23) | 9.13E-02 | 3.90E-17 | 71.54 |
| BIRC2 (Q13490) | 8.93 | 7.72 | 1.20 (1.01-1.39) | 9.38E-02 | 2.95E-16 | 73.59 |
| RRBP1 (Q9P2E9) | 14.65 | 13.91 | 0.74 (0.60-0.87) | 6.78E-02 | 4.09E-16 | 56.67 |
| LIPA1 (Q13136) | 10.55 | 14.29 | -3.74 (-4.47-(-3.00) | 3.69E-01 | 1.82E-15 | 33.08 |
| LTF (P02788) | 10.92 | 9.14 | 1.78 (1.42-1.42) | 1.82E-01 | 1.24E-13 | 51.03 |
| KCNN1 (Q92952) | 10.31 | 9.29 | 1.02 (0.79-1.25) | 1.15E-01 | 2.49E-13 | 43.08 |
| SH3L3 (Q9H299) | 9.81 | 8.42 | 1.39 (1.14-1.64) | 1.24E-01 | 7.30E-13 | 10.51 |
| COL6A1 (P12109) | 12.87 | 11.46 | 1.41 (1.09-1.72) | 1.59E-01 | 1.10E-12 | 58.46 |
| PTGES3 (Q15185) | 14.17 | 15.72 | -155 (-1.92-(-1.18)) | 1.85E-01 | 3.29E-12 | 28.97 |
| SRP14 (P37108) | 9.94 | 7.49 | 2.45 (2.03-2.86) | 2.03E-01 | 3.97E-12 | 56.92 |
| IL18 (Q14116) | 13.02 | 14.80 | -1.78 (-2.16-(-1.39)) | 1.91E-01 | 4.27E-12 | 58.46 |
| PDLIM1 (O00151) | 12.93 | 11.18 | 1.75 (1.33-2.17) | 2.09E-01 | 4.62E-12 | 40.51 |
| 1433Z (P63104) | 9.58 | 8.79 | 0.79 (0.60-0.99) | 9.91E-02 | 3.05E-11 | 45.90 |
| CASP10 (Q92851) | 9.34 | 10.76 | -1.43 (-1.79-(-1.06)) | 1.83E-01 | 3.30E-11 | 27.18 |
| FLII (Q13045) | 14.08 | 14.76 | -0.68 (-0.85-(-0.51)) | 8.62E-02 | 5.80E-11 | 71.28 |
| IKKA (O15111) | 11.35 | 13.11 | -1.75 (-2.19-(-1.30)) | 2.23E-01 | 6.70E-11 | 48.46 |
| TPP2 (P29144) | 13.64 | 14.50 | -0.86 (-1.08-(-0.64)) | 1.11E-01 | 7.31E-11 | 38.72 |
| DCD (P81605) | 9.87 | 8.24 | 1.63 (1.20-2.05) | 2.11E-01 | 7.43E-11 | 54.10 |
| RPIA (P49247) | 11.02 | 9.78 | 1.24 (0.91-1.57) | 1.64E-01 | 9.65E-11 | 33.08 |
| **Protein name (UniProt ID)** | **Mean expression RA** | **Mean expression HC** | **Difference in means (95% CI)** | **Standard error** | **p-value** | **% missing before imputation** |
| CALM (P62158) | 13.04 | 10.69 | 2.34 (1.72-2.96) | 3.11E-01 | 1.33E-10 | 5.64 |
| TBCA (O75347) | 12.27 | 13.82 | -1.54 (-1.95-(-1.13) | 2.05E-01 | 1.40E-10 | 35.90 |
| CLTC (Q00610) | 14.27 | 13.30 | 0.97 (0.72-1.22) | 1.26E-01 | 7.86E-10 | 57.44 |
| PLSL (P13796) | 15.11 | 15.97 | -0.86 (-1.10-(-0.62)) | 1.15E-01 | 3.45E-09 | 48.72 |
| CHI3L1 (P36222) | 13.69 | 15.46 | -1.77 (-2.28-(-1.27)) | 2.51E-01 | 7.18E-09 | 24.62 |
| ILF3 (Q12906) | 10.46 | 11.10 | -0.64 (-0.83-(-0.44) | 9.63E-02 | 7.54E-09 | 63.08 |
| PROF1 (P07737) | 12.56 | 11.07 | 1.48 (1.04-1.93) | 2.23E-01 | 2.35E-08 | 4.87 |
| NDUB4 (O95168) | 12.42 | 9.56 | 2.86 (2.24-3.49) | 2.97E-01 | 2.47E-08 | 43.85 |
| G3P (P04406) | 15.29 | 15.96 | -0.67 (-0.89-(-0.46) | 1.08E-01 | 3.64E-08 | 47.69 |
| MIF (P14174) | 10.46 | 9.54 | 0.92 (0.71-1.13) | 1.00E-01 | 4.32E-08 | 71.54 |
| PRS6A (P17980) | 16.56 | 15.48 | 1.08 (0.78-1.38) | 1.46E-01 | 4.63E-08 | 55.13 |
| APOM (O95445) | 16.30 | 17.02 | -0.72 (-0.94-(-0.50)) | 1.07E-01 | 4.74E-08 | 12.31 |
| ASPH (Q12797) | 11.53 | 13.27 | -1.74 (-2.26-(-1.23) | 2.53E-01 | 7.95E-08 | 41.54 |
| CD166 (Q13740) | 15.06 | 15.82 | -0.76 (-1.01-(-0.51)) | 1.24E-01 | 1.72E-07 | 50.77 |
| PLS3 (P13797) | 13.31 | 11.76 | 1.55 (1.03-2.07) | 2.58E-01 | 1.90E-07 | 38.21 |
| UTRN (P46939) | 14.50 | 15.41 | -0.92 (-1.18-(-0.65)) | 1.29E-01 | 2.07E-07 | 19.49 |
| SAA2 (P0DJI9) | 12.50 | 10.26 | 2.25 (1.59-2.90) | 3.17E-01 | 2.53E-07 | 65.90 |
| MAP1B (P46821) | 11.75 | 10.09 | 1.66 (1.06-2.26) | 3.01E-01 | 5.08E-07 | 58.46 |
| LBP (P18428) | 13.49 | 12.28 | 1.21 (0.81-1.60) | 1.94E-01 | 6.35E-07 | 0.51 |
| DPP3 (Q9NY33) | 12.38 | 13.53 | -1.16 (-1.46-(-0.85)) | 1.43E-01 | 6.51E-07 | 51.28 |
| ACTBL2 (Q562R1) | 11.94 | 10.77 | 1.17 (0.82-1.53) | 1.70E-01 | 7.46E-07 | 1.54 |
| CFH (P08603) | 17.63 | 17.05 | 0.59 (0.37-0.80) | 1.06E-01 | 9.08E-07 | 0.00 |
| RBM3 (P98179) | 17.50 | 18.42 | -0.92 (-1.24-(-0.60)) | 1.58E-01 | 1.15E-06 | 38.72 |
| LTBP1 (Q14766) | 13.45 | 12.36 | 1.09 (0.79-1.40) | 1.42E-01 | 1.39E-06 | 60.00 |
| ATP1A1 (P05023) | 12.78 | 13.78 | -1.00 (-1.35-(-0.65)) | 1.74E-01 | 1.66E-06 | 7.18 |
| CAT (P04040) | 8.73 | 8.25 | 0.48 (0.29-0.66) | 9.22E-02 | 2.01E-06 | 61.54 |
| SERPINA11 (Q86U17) | 8.04 | 8.60 | -0.56 (-0.78-(-0.35)) | 1.01E-01 | 2.09E-06 | 68.46 |
| ACOHC (P21399) | 16.34 | 17.32 | -0.98 (-1.36-(-0.60)) | 1.92E-01 | 2.48E-06 | 53.33 |
| IGKV1-17 (P01610) | 10.17 | 10.55 | -0.38 (-0.52-(-0.23)) | 7.32E-02 | 2.99E-06 | 73.85 |
| CAP1 (Q01518) | 10.14 | 9.03 | 1.11 (0.72-1.49) | 1.87E-01 | 3.26E-06 | 18.46 |
| PLXDC2 (Q6UX71) | 9.04 | 8.78 | 0.27 (0.16-0.37) | 5.30E-02 | 4.54E-06 | 68.21 |
| CFHR3 (Q02985) | 9.48 | 8.52 | 0.96 (0.64-1.28) | 1.52E-01 | 4.65E-06 | 66.67 |
| CRP (P02741) | 14.00 | 11.18 | 2.82 (1.79-3.84) | 4.98E-01 | 5.95E-06 | 10.51 |
| IL1RA (P18510) | 10.21 | 9.17 | 1.04 (0.64-1.44) | 1.96E-01 | 8.49E-06 | 15.90 |
| EF1A2 (Q05639) | 15.09 | 13.85 | 1.24 (0.85-1.63) | 1.84E-01 | 8.97E-06 | 73.85 |
| AKAP4 (Q5JQC9) | 10.43 | 9.33 | 1.09 (0.65-1.54) | 2.21E-01 | 1.17E-05 | 34.87 |
| PEPD (P12955) | 14.20 | 13.51 | 0.70 (0.40-0.99) | 1.49E-01 | 1.38E-05 | 56.67 |
| MAPK14 (Q16539) | 11.61 | 8.86 | 2.76 (1.85-3.66) | 4.21E-01 | 1.40E-05 | 53.59 |
| K2C1 (P04264) | 13.89 | 12.67 | 1.22 (0.79-1.64) | 2.00E-01 | 1.48E-05 | 56.41 |
| FUBP1 (Q96AE4) | 11.92 | 14.37 | -2.45 (-3.37-(-1.53)) | 4.39E-01 | 1.90E-05 | 64.10 |
| VARS1 (P26640) | 13.15 | 12.51 | 0.64 (0.39-0.90) | 1.25E-01 | 2.42E-05 | 65.38 |
| INVS (Q9Y283) | 13.18 | 12.18 | 1.00 (0.56-1.45) | 2.23E-01 | 3.11E-05 | 54.87 |
| APOC1 (P02654) | 15.78 | 16.94 | -1.16 (01.64-(-0.68)) | 2.35E-01 | 3.13E-05 | 0.51 |
| IGKV3-15 (P04207) | 9.85 | 10.90 | -1.05 (-1.46-(-0.65)) | 1.91E-01 | 3.26E-05 | 77.69 |
| IC1 (P05155) | 17.70 | 19.35 | -1.64 (-2.32-(-0.97)) | 3.28E-01 | 4.27E-05 | 0.00 |
| TMSB10 (P63313) | 19.18 | 18.55 | 0.63 (0.34-0.92) | 1.44E-01 | 4.86E-05 | 75.13 |
| WDR1 (O75083) | 10.67 | 6.78 | 3.89 (2.35-5.43) | 7.28E-01 | 5.51E-05 | 65.64 |
| COL6A3 (P12111) | 15.76 | 14.93 | 0.84 (0.49-1.18) | 1.66E-01 | 6.15E-05 | 42.05 |
| **Protein name (UniProt ID)** | **Mean expression RA** | **Mean expression HC** | **Difference in means (95% CI)** | **Standard error** | **p-value** | **% missing before imputation** |
| IGF1 (P05019) | 8.97 | 10.31 | -1.34 (-1.88-(-0.80)) | 2.56E-01 | 7.23E-05 | 78.97 |
| TXNDC5 (Q8NBS9) | 16.10 | 15.35 | 0.75 (0.41-1.10) | 1.71E-01 | 7.39E-05 | 38.21 |
| LAMP2 (P13473) | 9.27 | 8.53 | 0.75 (0.41-1.08) | 1.62E-01 | 8.08E-05 | 28.21 |
| APOB (P04114) | 18.20 | 18.92 | -0.72 (-1.05-(-0.39)) | 1.61E-01 | 8.25E-05 | 0.00 |
| UGGT1 (Q9NYU2) | 9.89 | 8.23 | 1.66 (0.97-2.35) | 3.27E-01 | 9.34E-05 | 69.74 |
| ALDOA (P04075) | 9.88 | 11.69 | -1.81 (-2.62-(-0.99)) | 3.94E-01 | 1.38E-04 | 84.62 |
| APOC2 (P02655) | 15.81 | 17.06 | -1.25 (-1.83-(-0.67)) | 2.83E-01 | 1.47E-04 | 0.00 |
| PFKP (Q01813) | 7.48 | 6.39 | 1.09 (0.56-1.63) | 2.64E-01 | 1.66E-04 | 26.41 |
| TIF1B (Q13263) | 10.70 | 10.07 | 0.62 (0.31-0.93) | 1.56E-01 | 1.91E-04 | 62.31 |
| TRAF2 (Q12933) | 11.35 | 10.69 | 0.66 (0.33-0.99) | 1.63E-01 | 2.21E-04 | 36.92 |
| C1R (P00736) | 15.00 | 14.47 | 0.53 (0.27-0.80) | 1.30E-01 | 2.45E-04 | 0.00 |
| C11orf54 (Q9H0W9) | 17.25 | 16.49 | 0.76 (0.37-1.15) | 1.95E-01 | 2.46E-04 | 49.23 |
| PKP3 (Q9Y446) | 14.06 | 12.46 | 1.59 (0.86-2.33) | 3.47E-01 | 2.70E-04 | 43.85 |
| DYNC1H1 (Q14204) | 19.05 | 18.78 | -0.72 (-1.10-(-0.35)) | 1.88E-01 | 2.73E-04 | 25.64 |
| SERPINH1 (P50454) | 20.06 | 19.73 | 0.33 (0.16-0.50) | 8.69E-02 | 2.79E-04 | 32.31 |
| DAB2IP (Q5VWQ8) | 7.32 | 10.93 | -3.61 (-5.34-(-1.87)) | 8.26E-01 | 3.69E-04 | 33.33 |
| TCEA2 (Q15560) | 10.49 | 10.71 | -0.23 (-0.35-(-0.10)) | 6.07E-02 | 4.07E-04 | 47.18 |
| REG1A (P05451) | 11.16 | 10.74 | 0.42 (0.20-0.63) | 1.04E-01 | 4.26E-04 | 43.33 |
| CFHR5 (Q9BXR6) | 9.80 | 9.13 | 0.68 (0.33-1.02) | 1.69E-01 | 4.78E-04 | 41.03 |
| SODE (P08294) | 8.70 | 7.97 | 0.73 (0.33-1.13) | 2.01E-01 | 5.15E-04 | 50.77 |
| FOXO1 (Q12778) | 9.70 | 9.07 | 0.63 (0.29-0.96) | 1.68E-01 | 5.48E-04 | 64.10 |
| CAPN1 (P07384) | 11.17 | 11.66 | -0.49 (-0.77-(-0.22)) | 1.37E-01 | 7.02E-04 | 54.87 |
| APOA4 (P06727) | 19.78 | 18.42 | 1.36 (0.62-2.10) | 3.60E-01 | 8.00E-04 | 13.33 |
| APMAP (Q9HDC9) | 11.26 | 11.92 | -0.65 (-1.00-(-0.31)) | 1.68E-01 | 8.44E-04 | 57.95 |
| VGFR1 (P17948) | 13.56 | 13.03 | 0.53 (0.24-0.83) | 1.46E-01 | 8.80E-04 | 43.85 |
| LYSC (P61626) | 12.48 | 11.05 | 1.42 (0.68-2.17) | 3.52E-01 | 9.98E-04 | 4.10 |
| PK3CD (O00329) | 9.17 | 7.36 | 1.81 (0.80-2.82) | 4.87E-01 | 1.13E-03 | 38.72 |
| PSMD1 (Q99460) | 13.90 | 12.60 | 1.30 (0.55-2.04) | 3.64E-01 | 1.28E-03 | 33.08 |
| PGK1 (P00558) | 12.05 | 10.87 | 1.18 (0.51-1.85) | 3.27E-01 | 1.34E-03 | 38.21 |
| DBH (P09172) | 9.66 | 10.40 | -0.74 (-1.17-(-0.32)) | 2.08E-01 | 1.38E-03 | 35.64 |
| CPX3 (P22352) | 13.10 | 13.68 | -0.59 (-0.94-(-0.23)) | 1.76E-01 | 1.62E-03 | 0.00 |
| COTL1 (Q14019) | 11.20 | 10.51 | 0.70 (0.30-1.09) | 1.90E-01 | 1.63E-03 | 48.46 |
| LMNB1 (P20700) | 12.85 | 13.74 | -0.90 (-1.40-(-0.39)) | 2.36E-01 | 1.70E-03 | 21.03 |
| TNF (P01375) | 11.00 | 9.46 | 1.54 (0.68-2.40) | 4.00E-01 | 1.76E-03 | 40.77 |
| ECHA (P40939) | 9.73 | 10.28 | -0.55 (-0.88-(-0.21)) | 1.67E-01 | 1.75E-03 | 56.92 |
| CALU (O43852) | 11.38 | 10.64 | 0.74 (0.31-1.16) | 2.01E-01 | 1.84E-03 | 47.69 |
| PROP (P27918) | 12.40 | 11.46 | 0394 (0.39-1.49) | 2.66E-01 | 2.05E-03 | 0.00 |
| RANG (P43487) | 8.96 | 8.62 | 0.34 (0.13-0.54) | 9.93E-02 | 2.06E-03 | 67.18 |
| HBS1L (Q9Y450) | 13.62 | 14.13 | -0.51 (-0.82-(-0.20)) | 1.51E-01 | 2.12E-03 | 19.49 |
| C4B (P0C0L5) | 16.09 | 17.02 | -0.93 (-1.50-(-0.36)) | 2.78E-01 | 2.18E-03 | 6.92 |
| CATD (P07339) | 19.85 | 16.67 | 3.18 (1.30-5.06) | 8.95E-01 | 2.35E-03 | 54.62 |
| MAPK8 (P45983) | 10.38 | 11.16 | -0.78 (-1.27-(-0.29)) | 2.42E-01 | 2.48E-03 | 54.36 |
| TCPH (Q99832) | 14.73 | 13.74 | 0.99 (0.41-1.56) | 2.70E-01 | 2.55E-03 | 51.28 |
| AHNAK (Q09666) | 19.61 | 19.14 | 0.46 (0.17-0.75) | 1.44E-01 | 2.91E-03 | 44.36 |
| GARS (P41250) | 14.54 | 13.96 | 0.58 (0.22-0.94) | 1.74E-01 | 3.03E-03 | 65.13 |
| XRCC5 (P13010) | 9.06 | 9.92 | -0.85 (-1.38-(-0.32)) | 2.56E-01 | 3.09E-03 | 15.13 |
| MDHM (P40926) | 13.11 | 12.82 | 0.29 (0.10-0.49) | 9.49E-02 | 3.09E-03 | 67.69 |
| MAPK3 (P27361) | 8.88 | 9.53 | -0.65 (-1.07-(-0.24)) | 2.02E-01 | 3.20E-03 | 65.38 |
| PYGL (P06737) | 13.86 | 14.17 | -0.31 (-0.50-(-0.11)) | 9.42E-02 | 3.42E-03 | 69.74 |
|  |  |  |  |  |  |  |
|  |  |  |  |  |  |  |
| **Protein name (UniProt ID)** | **Mean expression RA** | **Mean expression HC** | **Difference in means (95% CI)** | **Standard error** | **p-value** | **% missing before imputation** |
| MYH13 (Q9UKX3) | 8.99 | 9.67 | -0.68 (-1.12-(-0.24)) | 2.15E-01 | 3.45E-03 | 64.10 |
| MYLK (Q15746) | 13.05 | 12.45 | 0.59 (0.22-0.96) | 1.77E-01 | 3.66E-03 | 54.36 |
| NOE1 (Q99784) | 10.30 | 9.98 | 0.32 (0.11-0.52) | 9.90E-02 | 4.43E-03 | 51.28 |
| PARD3 (Q8TEW0) | 11.80 | 10.39 | 1.41 (0.49-2.33) | 4.39E-01 | 4.62E-03 | 33.59 |
| 1433F (Q04917) | 9.12 | 8.17 | 0.95 (0.33-1.57) | 2.98E-01 | 4.89E-03 | 72.05 |
| MICU1 (Q9BPX6) | 9.76 | 10.60 | -0.85 (-1.41-(-0.28)) | 2.73E-01 | 5.47E-03 | 42.82 |
| CLU (P10909) | 17.89 | 17.53 | 0.35 (0.11-0.60) | 1.21E-01 | 5.66E-03 | 0.00 |
| GGH (Q92820) | 9.99 | 9.36 | 0.63 (0.20-1.06) | 2.11E-01 | 5.72E-03 | 32.56 |
| HEP2 (P05546) | 18.27 | 17.85 | 0.42 (0.13-0.71) | 1.44E-01 | 5.75E-03 | 0.00 |
| NAA25 (Q14CX7) | 12.61 | 11.15 | 1.46 (0.49-2.43) | 4.55E-01 | 5.81E-03 | 48.97 |
| FETUB (Q9UGM5) | 12.22 | 11.60 | 0.62 (0.19-1.06) | 2.13E-01 | 6.61E-03 | 1.79 |
| UBP14 (P54578) | 14.18 | 14.54 | -0.36 (-0.61-(-0.11)) | 1.23E-01 | 6.79E-03 | 57.69 |
| GLOD4 (Q9HC38) | 13.54 | 14.11 | -0.57 (-0.99-(-0.16)) | 2.06E-01 | 6.83E-03 | 41.28 |
| RPS3 (P23396) | 10.91 | 9.28 | 1.63 (0.52-2.75) | 5.25E-01 | 6.96E-03 | 21.79 |
| IGHV3-23 (P01776) | 18.57 | 17.08 | 1.50 (0.44-2.55) | 5.06E-01 | 7.65E-03 | 57.44 |
| F13B (P05160) | 11.78 | 11.35 | 0.42 (0.1-0.72) | 1.37E-01 | 7.69E-03 | 56.92 |
| APOC3 (P02656) | 16.44 | 17.38 | -0.94 (-1.61-(-0.28)) | 3.22E-01 | 7.80E-03 | 0.00 |
| MMP9 (P14780) | 10.33 | 9.69 | 0.64 (0.18-1.09) | 2.26E-01 | 7.96E-03 | 26.67 |
| PON1 (P27169) | 16.15 | 16.73 | -0.58 (-0.99-(-0.16)) | 1.97E-01 | 9.05E-03 | 0.00 |
| MON2 (Q7Z3U7) | 18.80 | 18.00 | 0.80 (0.21-1.39) | 2.87E-01 | 9.68E-03 | 8.21 |
| SCFD1 (Q8WVM8) | 15.68 | 13.50 | 2.18 (0.61-3.75) | 7.35E-01 | 9.90E-03 | 34.10 |
| PTGDS (P41222) | 10.00 | 10.44 | -0.43 (-0.75-(-0.11)) | 1.53E-01 | 1.03E-02 | 72.31 |
| AKT1 (P31749) | 9.64 | 9.15 | 0.49 (0.12-0.85) | 1.75E-01 | 1.08E-02 | 67.44 |
| HNRPNPM (P52272) | 16.46 | 16.24 | 0.21 (0.05-0.38) | 8.19E-02 | 1.10E-02 | 68.46 |
| AACT (P01011) | 20.94 | 20.40 | 0.53 (0.13-0.93) | 1.92E-01 | 1.11E-02 | 0.00 |
| SND1 (Q7KZF4) | 16.88 | 15.53 | 1.34 (0.34-2.35) | 4.76E-01 | 1.13E-02 | 24.36 |
| NFKB1 (P19838) | 15.14 | 14.13 | 1.01 (0.25-1.78) | 3.75E-01 | 1.15E-02 | 10.77 |
| CA2 (P00918) | 8.68 | 8.18 | 0.50 (0.12-0.88) | 1.86E-01 | 1.19E-02 | 19.49 |
| MMRN1 (Q13201) | 12.78 | 12.25 | 0.53 (0.13-0.94) | 1.95E-01 | 1.19E-02 | 63.85 |
| CFHR1 (Q03591) | 12.05 | 10.81 | 1.24 (0.31-2.17) | 4.38E-01 | 1.20E-02 | 11.54 |
| MLKL (Q8NB16) | 11.47 | 11.15 | 0.32 (0.08-0.57) | 1.17E-01 | 1.22E-02 | 26.67 |
| C9 (P02748) | 17.03 | 16.47 | 0.56 (0.13-0.99) | 2.07E-01 | 1.23E-02 | 0.00 |
| PTBP1 (P26599) | 11.69 | 12.16 | -0.47 (-0.83-(-0.18)) | 1.82E-01 | 1.25E-02 | 58.21 |
| ADHX (P11766) | 16.66 | 16.40 | 0.26 (0.06-0.46) | 9.78E-02 | 1.37E-02 | 56.67 |
| GDIR2 (P52566) | 14.53 | 13.67 | 0.86 (0.18-1.54) | 3.31E-01 | 1.48E-02 | 52.56 |
| DHX15 (O43143) | 11.77 | 13.14 | -1.37 (-2.45-(-0.30)) | 5.01E-01 | 1.57E-02 | 57.69 |
| MRC1 (P22897) | 12.35 | 12.94 | -0.59 (-1.05-(-0.13)) | 2.17E-01 | 1.60E-02 | 43.33 |
| NGAL (P80188) | 10.65 | 10.86 | -0.21 (-0.38-(-0.04)) | 8.40E-02 | 1.62E-02 | 76.15 |
| MASP1 (P48740) | 11.02 | 11.60 | -0.58 (-1.05-(-0.11)) | 2.29E-01 | 1.73E-02 | 6.15 |
| F11 (P03951) | 12.02 | 11.00 | 1.02 (0.20-1.84) | 3.91E-01 | 1.75E-02 | 2.82 |
| AMPN (P15144) | 12.58 | 11.79 | 0.80 (0.16-1.44) | 3.00E-01 | 1.75E-02 | 41.28 |
| THIM (P42765) | 12.00 | 11.66 | 0.34 (0.06-0.63) | 1.36E-01 | 2.13E-02 | 45.38 |
| PF4 (P02776) | 12.66 | 12.15 | 0.51 (0.08-0.94) | 2.10E-01 | 2.14E-02 | 8.46 |
| SAA1 (P0DJI8) | 11.21 | 9.88 | 1.33 (0.21-2.46) | 5.45E-01 | 2.25E-02 | 26.92 |
| CTND1 (O60716) | 11.38 | 10.27 | 1.10 (0.17-2.04) | 4.47E-01 | 2.37E-02 | 37.69 |
| COL1A1 (P02452) | 11.31 | 10.67 | 0.64 (0.10-1.18) | 2.59E-01 | 2.39E-02 | 61.79 |
| CD163 (Q86VB7) | 10.32 | 9.79 | 0.54 (0.07-1.00) | 2.23E-01 | 2.46E-02 | 32.05 |
| **Protein name (UniProt ID)** | **Mean expression RA** | **Mean expression HC** | **Difference in means (95% CI)** | **Standard error** | **p-value** | **% missing before imputation** |
| CACNA1D (Q01668) | 9.82 | 9.20 | 0.62 (0.09-1.16) | 2.48E-01 | 2.51E-02 | 61.79 |
| PRDX3 (P30048) | 15.17 | 12.28 | 2.89 (0.42-5.36) | 1.15E-01 | 2.53E-02 | 13.59 |
| FLNB (O75369) | 16.46 | 16.84 | -0.38 (-0.71-(-0.05) | 1.56E-01 | 2.57E-02 | 52.82 |
| SAMP (P02743) | 16.78 | 16.26 | 0.52 (0.07-0.97) | 2.17E-01 | 2.62E-02 | 0.26 |
| MAP2K3 (P46734) | 10.73 | 10.41 | 0.31 (0.04-0.59) | 1.37E-01 | 2.63E-02 | 68.97 |
| CAND1 (Q86VP6) | 14.37 | 14.83 | -0.46 (-0.87-(-0.01)) | 1.95E-01 | 2.94E-02 | 48.72 |
| GP1BA (P07359) | 13.26 | 12.66 | 0.60 (0.07-1.14) | 2.59E-01 | 2.95E-02 | 8.21 |
| APOD (P05090) | 17.27 | 17.65 | -0.38 (-0.72-(-0.04)) | 1.69E-02 | 2.97E-02 | 0.00 |
| A1AG1 (P02763) | 19.57 | 18.90 | 0.68 (0.07-1.28) | 2.97E-01 | 3.04E-02 | 0.00 |
| RPS4X (P62701) | 13.40 | 13.08 | 0.32 (0.03-0.61) | 1.39E-01 | 3.30E-02 | 12.56 |
| RBP4 (P02753) | 17.33 | 17.70 | -0.37 (-0.70-(-0.03)) | 1.65E-01 | 3.35E-02 | 0.00 |
| LCAT (P04180) | 13.23 | 12.99 | 0.24 (0.02-0.47) | 1.10 E-01 | 3.36E-02 | 0.00 |
| VWF (P04275) | 14.45 | 14.60 | -0.15 (-0.28-(-0.01)) | 6.48E-02 | 3.38E-02 | 54.87 |
| HSPD1 (P10809) | 15.50 | 16.02 | -0.53 (-1.01-(-0.04)) | 2.33E-01 | 3.44E-02 | 48.21 |
| CAMP (P49913) | 10.98 | 10.45 | 0.53 (0.04-1.02) | 2.38E-01 | 3.64E-02 | 20.25 |
| VTN (P04004) | 18.83 | 18.45 | 0.37 (0.02-0.72) | 1.68E-01 | 3.68E-02 | 0.00 |
| SELENOP (P49908) | 11.27 | 11.70 | -0.42 (-0.82-(-0.03)) | 1.94E-01 | 3.69E-02 | 6.92 |
| C1S (P09871) | 15.62 | 15.29 | 0.32 (0.02-0.63) | 1.51E-01 | 4.05E-02 | 0.00 |
| HBB (P68871) | 17.82 | 16.92 | 0.90 (0.04-1.75) | 4.09E-01 | 4.09E-02 | 0.00 |
| ENOG (P09104) | 16.42 | 16.57 | -0.15 (-0.29-(-0.00)) | 7.20E-02 | 4.32E-02 | 55.90 |
| MINPP1 (Q9UNW1) | 11.33 | 10.96 | 0.37 (0.01-0.73) | 1.71E-01 | 4.39E-02 | 53.33 |
| K2C1B (Q7Z794) | 11.75 | 12.13 | -0.38 (-0.74-(-0.01)) | 1.83E-01 | 4.43E-02 | 41.03 |
| YCF51 (P49327) | 17.85 | 17.39 | 0.45 (0.01-0.90) | 2.13E-01 | 4.50E-02 | 18.97 |
| A2AP (P08697) | 16.25 | 16.69 | -0.44 (-0.87-(-0.01)) | 2.05E-01 | 4.68E-02 | 0.00 |
| EPIPL (P58107) | 15.13 | 15.40 | -0.27 (-0.54-(-0.00)) | 1.32E-01 | 4.82E-02 | 64.36 |

Please note: only abbreviated protein names have been included in this table for brevity. Please access <https://www.uniprot.org/> for full protein names and descriptions.

*Supplementary Table S2. Proteins measured before treatment with etanercept significantly associated with DAS28 remission (<2.6) at 3 months, univariate analysis adjusted for potential confounders.*

| **Variable** | **OR_adj_ (95% CI)** | **p-value** | **Adjusted p-value** | **% missing before imputation** |
| --- | --- | --- | --- | --- |
| COL6A2 (P12110) | 2.24 (1.45 – 3.45) | 2.65E-04 | 3.64E-02 | 60.77 |
| EHD1 (Q9H4M9) | 0.58 (0.43 – 0.78) | 3.37E-04 | 3.64E-02 | 72.82 |
| TCPH (Q99832) | 0.21 (0.08 – 0.51) | 6.63E-04 | 4.78E-02 | 51.28 |

Abbreviations: Adjusted (adj), collagen α-2(VI) chain (COL6A2), confidence intervals (CI), EH domain-containing protein 1 (EHD1), odds ratio (OR), T-complex protein 1 subunit η (TCPH).

*Supplementary Table S3. Proteins measured before treatment with etanercept significantly associated with DAS28 remission (<2.6) at 3 months, multivariable model.*

| **Variable** | **OR_adj_ (95% CI)** | **p-value** | **% missing before imputation** |
| --- | --- | --- | --- |
| COL6A2 (P12110) | 1.23 (0.66 – 2.27) | 0.51 | 60.77 |
| EHD1 (Q9H4M9) | 0.70 (0.46 – 1.04) | 0.09 | 72.82 |
| TCPH (Q99832) | 0.32 (0.11 – 0.85) | 2.91E-02* | 51.28 |
| Age at baseline | 0.97 (0.93 – 1.00) | 0.06 | N/A |
| Disease duration | 1.00 (0.96 – 1.04) | 0.83 | N/A |
| BMI | 0.96 (0.90 – 1.02) | 0.20 | N/A |
| Seropositivity (RF/ACPA) | 2.83 (1.19 – 7.14) | 2.20E-02* | N/A |
| Baseline DAS28 | 1.52 (0.96 – 2.53) | 0.09 | N/A |
| Male sex | 1.68 (0.68 – 4.21) | 0.26 | N/A |
| Concurrent csDMARD | 1.17 (0.45 – 3.17) | 0.74 | N/A |
| Systemic steroid use | 0.51 (0.19 – 1.27) | 0.16 | N/A |
| Comorbidity | 1.27 (0.52 – 3.10) | 0.59 | N/A |

Abbreviations: Adjusted (adj), anti-citrullinated peptide antigen (ACPA), body mass index (BMI), collagen α-2(VI) chain (COL6A2), confidence intervals (CI), conventional synthetic disease-modifying anti-rheumatic drug (csDMARD), Disease Activity Score of 28 Joints (DAS28), EH domain-containing protein 1 (EHD1), odds ratio (OR), rheumatoid factor (RF), T-complex protein 1 subunit η (TCPH).

*Supplementary Table S4. Proteins measured before treatment with etanercept significantly associated with DAS28 at 3 months, univariate analysis adjusted for potential confounders.*

| **Variable** | **β-coefficient_adj_ (95% CI)** | **p-value** | **Adjusted p-value** | **% missing before imputation** |
| --- | --- | --- | --- | --- |
| EHD1 (Q9H4M9) | 0.31 (0.15 – 0.48) | 2.85E-04 | 3.11E-02 | 72.82 |
| TCPH (Q99832) | 0.11 (0.05 – 0.16) | 2.88E-04 | 3.11E-02 | 51.28 |

Abbreviations: Adjusted (adj), confidence intervals (CI), EH domain-containing protein 1 (EHD1), T-complex protein 1 subunit η (TCPH).

*Supplementary Table S5. Proteins measured before treatment with etanercept significantly associated with DAS28 at 3 months, multivariable model.*

| **Variable** | **β-coefficient_adj_ (95% CI)** | **p-value** | **% missing before imputation** |
| --- | --- | --- | --- |
| EHD1 (Q9H4M9) | 0.21 (0.05 – 0.37) | 9.49E-03* | 72.82 |
| TCPH (Q99832) | 0.62 (0.16 – 1.08) | 9.59E-03* | 51.28 |
| Age at baseline | 0.02 (0.00 – 0.04) | 0.02* | N/A |
| Disease duration | -0.01 (-0.03 – 0.1) | 0.34 | N/A |
| BMI | 0.05 (0.02 – 0.08) | 3.30E-03* | N/A |
| Seropositivity (RF/ACPA) | -0.28 (-0.75 – 0.18) | 0.23 | N/A |
| Baseline DAS28 | 0.05 (-0.19 – 0.29) | 6.75 | N/A |
| Male sex | -0.29 (-0.77 – 0.19) | 0.24 | N/A |
| Concurrent csDMARD | -0.03 (-0.56 – 0.50) | 0.92 | N/A |
| Systemic steroid use | 0.68 (0.19 – 1.16) | 7.36E-03* | N/A |
| Comorbidity | -0.10 (-0.57 – 0.37) | 0.68 | N/A |

Abbreviations: Adjusted (adj), anti-citrullinated peptide antigen (ACPA), body mass index (BMI), confidence intervals (CI), conventional synthetic disease-modifying anti-rheumatic drug (csDMARD), EH domain-containing protein 1 (EHD1), rheumatoid factor (RF), T-complex protein 1 subunit η (TCPH).

*Supplementary Table S6. Proteins measured before treatment with etanercept significantly associated with CRP at 6 months, univariate analysis adjusted for potential confounders.*

| **Variable** | **β-coefficient_adj_ (95% CI)** | **p-value** | **Adjusted p-value** | **% missing before imputation** |
| --- | --- | --- | --- | --- |
| SELENOP (P49908) | -0.01 (-0.02 – (-0.01)) | 2.35E-04 | 4.10E-02 | 6.92 |
| MAP2K3 (P46734) | 0.01 (0.00 – 0.02) | 5.69E-04 | 4.10E-02 | 68.97 |
| CLTC (Q00610) | 0.01 (0.00 – 0.02) | 7.93E-04 | 4.28E-02 | 57.44 |

Abbreviations: Adjusted (adj), clathrin heavy chain 1 (CLTC), confidence intervals (CI), dual specificity mitogen-activated protein kinase kinase 3 (MAP2K3), selenoprotein P (SELENOP).

*Supplementary Table S7. Proteins measured before treatment with etanercept significantly associated with CRP at 6 months, multivariable model.*

| **Variable** | **β-coefficient_adj_ (95% CI)** | **p-value** | **% missing before imputation** |
| --- | --- | --- | --- |
| SELENOP (P49908) | -5.68 (-9.19 – (-2.17)) | 1.87E-03* | 6.92 |
| MAP2K3 (P46734) | 7.85 (3.49 – 12.22) | 5.79E-03* | 68.97 |
| CLTC (Q00610) | 4.76 (0.77 – 8.75) | 2.08E-02* | 57.44 |
| Age at baseline | 0.19 (-0.08 – 0.46) | 0.17 | N/A |
| Disease duration | -0.26 (-0.55 – 0.02) | 0.07 | N/A |
| BMI | 0.23 (-0.20 – 0.65) | 0.29 | N/A |
| Seropositivity (RF/ACPA) | -0.68 (-6.85 – 5.49) | 0.83 | N/A |
| Baseline DAS28 | -1.64 (-4.93 – 1.65) | 0.33 | N/A |
| Male sex | -0.65 (-7.14 – 5.84) | 0.85 | N/A |
| Concurrent csDMARD | -0.22 (-7.27 – 6.84) | 0.95 | N/A |
| Systemic steroid use | 4.93 (-1.64 – 11.49) | 0.14 | N/A |
| Comorbidity | -0.38 (-6.74 – 5.98) | 0.91 | N/A |

Abbreviations: Adjusted (adj), anti-citrullinated peptide antigen (ACPA), body mass index (BMI), clathrin heavy chain 1 (CLTC), confidence intervals (CI), conventional synthetic disease-modifying anti-rheumatic drug (csDMARD), Disease Activity Score of 28 Joints (DAS28), dual specificity mitogen-activated protein kinase kinase 3 (MAP2K3), rheumatoid factor (RF), selenoprotein P (SELENOP).

*Supplementary Table S8. Proteins measured after 3 months of treatment with etanercept significantly associated with VAS-GH at 6 months, univariate analysis adjusted for potential confounders.*

| **Variable** | **β-coefficient_adj_ (95% CI)** | **p-value** | **Adjusted p-value** | **% missing before imputation** |
| --- | --- | --- | --- | --- |
| ASPH (Q12797) | -0.01 (-0.02 – (-0.01)) | 1.45E-04 | 3.14E-02 | 41.54 |

Abbreviations: Adjusted (adj), aspartyl/asparaginyl β-hydroxylase (ASPH), confidence intervals (CI).

*Supplementary Table S9. Proteins measured after 3 months of treatment with etanercept significantly associated with CRP at 6 months, univariate analysis adjusted for potential confounders.*

| **Variable** | **β-coefficient_adj_ (95% CI)** | **p-value** | **Adjusted p-value** | **% missing before imputation** |
| --- | --- | --- | --- | --- |
| SAA1 (P0DJI8) | 0.03 (0.01 – 0.04) | 9.79E-05 | 1.06E-02 | 26.92 |
| A1AG1 (P02763) | 0.02 (0.01 – 0.03) | 3.42E-04 | 1.44E-02 | 0.00 |
| ASPH (Q12797) | -0.01 (-0.02 – (-0.01)) | 3.58E-04 | 1.44E-02 | 41.54 |
| MAP2K3 (P46734) | 0.01 (0.00 – 0.02) | 3.86E-04 | 1.44E-02 | 68.97 |
| AACT (P01011) | 0.01 (0.01 – 0.02) | 3.99E-04 | 1.44E-02 | 0.00 |
| MYLK (Q15746) | 0.01 (0.00 – 0.01) | 5.06E-04 | 1.56E-02 | 54.36 |
| CFHR5 (Q9BXR6) | 0.01 (0.00 – 0.02) | 7.57E-04 | 2.04E-02 | 41.03 |
| C9 (P02748) | 0.01 (0.00 – 0.02) | 1.10E-04 | 2.64E-02 | 0.00 |

Abbreviations: α-1-acid glycoprotein 1 (A1AG1), α-1-antichymotrypsin (AACT), adjusted (adj), aspartyl/asparaginyl β-hydroxylase (ASPH), complement component C9 (C9), complement factor H-related protein 5 (CFHR5), confidence intervals (CI), dual specificity mitogen-activated protein kinase kinase 3 (MAP2K3), myosin light chain kinase, smooth muscle (MYLK), serum amyloid A-1 protein (SAA1).

*Supplementary Table S10. Proteins measured after 3 months of treatment with etanercept significantly associated with CRP at 6 months, multivariable model.*

| **Variable** | **β-coefficient_adj_ (95% CI)** | **p-value** | **% missing before imputation** |
| --- | --- | --- | --- |
| SAA1 (P0DJI8) | 3.24 (1.22 – 5.26) | 2.00E-03* | 26.92 |
| A1AG1 (P02763) | 2.87 (-0.14 – 5.89) | 0.06 | 0.00 |
| ASPH (Q12797) | -3.17 (-6.39 – 0.05) | 0.06 | 41.54 |
| MAP2K3 (P46734) | 6.30 (1.72 – 10.89) | 7.93E-03* | 68.97 |
| AACT (P01011) | 0.82 (-5.28 – 6.93) | 0.79 | 0.00 |
| MYLK (Q15746) | 8.92 (1.13 – 16.72) | 2.64E-02* | 54.36 |
| CFHR5 (Q9BXR6) | 2.59 (-1.28 – 6.46) | 0.19 | 41.03 |
| C9 (P02748) | -4.12 (-10.36 – 2.12) | 0.20 | 0.00 |
| Age at baseline | 0.16 (-0.09 – 0.42) | 0.22 | N/A |
| Disease duration | -0.01 (-0.27 – 0.26) | 0.97 | N/A |
| BMI | -0.12 (-0.52 – 0.29) | 0.57 | N/A |
| Seropositivity (RF/ACPA) | -0.61 (-6.60 – 5.38) | 0.84 | N/A |
| Baseline DAS28 | 0.10 (-2.88 – 3.08) | 0.95 | N/A |
| Male sex | -0.88 (-7.15 – 5.38) | 0.78 | N/A |
| Concurrent csDMARD | -1.08 (-7.87 – 5.71) | 0.76 | N/A |
| Systemic steroid use | -6.01 (-12.26 – 0.24) | 0.06 | N/A |
| Comorbidity | -0.56 (-6.38 – 5.25) | 0.85 | N/A |

Abbreviations: α-1-acid glycoprotein 1 (A1AG1), α-1-antichymotrypsin (AACT), adjusted (adj), anti-citrullinated peptide antigen (ACPA), aspartyl/asparaginyl β-hydroxylase (ASPH), body mass index (BMI), complement component C9 (C9), complement factor H-related protein 5 (CFHR5), confidence intervals (CI), conventional synthetic disease-modifying anti-rheumatic drug (csDMARD), Disease Activity Score of 28 Joints (DAS28), dual specificity mitogen-activated protein kinase kinase 3 (MAP2K3), myosin light chain kinase, rheumatoid factor (RF), smooth muscle (MYLK), serum amyloid A-1 protein (SAA1).

*Supplementary Table S11. Proteins measured after 3 months of treatment with etanercept significantly associated with DAS28 at 6 months, univariate analysis adjusted for potential confounders.*

| **Variable** | **β-coefficient_adj_ (95% CI)** | **p-value** | **Adjusted p-value** | **% missing before imputation** |
| --- | --- | --- | --- | --- |
| CRP (P02741) | 0.38 (0.18 – 0.57) | 2.23E-04 | 2.85E-02 | 10.51 |
| C9 (P0748) | 0.15 (0.07 – 0.22) | 2.64E-04 | 2.85E-02 | 0.00 |

Abbreviations: Adjusted (adj), C-reactive protein (CRP), complement component C9 (C9), confidence intervals (CI).

*Supplementary Table S12. Proteins measured after 3 months of treatment with etanercept significantly associated with DAS28 at 6 months, multivariable model.*

| **Variable** | **β-coefficient_adj_ (95% CI)** | **p-value** | **% missing before imputation** |
| --- | --- | --- | --- |
| CRP (P02741) | 0.16 (0.01 – 0.30) | 3.93E-02* | 10.51 |
| C9 (P02748) | 0.38 (0.01 – 0.76) | 4.74E-02* | 0.00 |
| Age at baseline | 0.01 (-0.01 – 0.04) | 0.28 | N/A |
| Disease duration | -0.01 (-0.04 – 0.01) | 0.27 | N/A |
| BMI | 0.05 (0.01 – 0.09) | 6.96E-03* | N/A |
| Seropositivity (RF/ACPA) | -0.28 (-0.80 – 0.23) | 0.29 | N/A |
| Baseline DAS28 | -0.04 (-0.30 – 0.23) | 0.79 | N/A |
| Male sex | -0.34 (-0.89 – 0.21) | 0.23 | N/A |
| Concurrent csDMARD | 0.18 (-0.41 – 0.76) | 0.56 | N/A |
| Systemic steroid use | 0.55 (0.03 – 1.07) | 4.12E-02* | N/A |
| Comorbidity | 0.32 (-0.20 – 0.83) | 0.23 | N/A |

Abbreviations: Adjusted (adj), anti-citrullinated peptide antigen (ACPA), body mass index (BMI), C-reactive protein (CRP), complement component C9 (C9), confidence intervals (CI), conventional synthetic disease-modifying anti-rheumatic drug (csDMARD), Disease Activity Score of 28 Joints (DAS28), rheumatoid factor (RF).

*Supplementary Table S13. Validation of proteins predictive of RA disease outcomes after treatment with etanercept, adjusted in multivariable models as per Table 2 in main text.*

| **Protein** | **Protein measurement time point** | **Outcome measure time point** | **OR_adj_ (95% CI)** | **Adjusted p-value** |
| --- | --- | --- | --- | --- |
| *DAS28 remission (<2.6)* | | | | |
| TCPH (Q99832) | Baseline | 3 months | 0.06 (0.00 – 0.50) | 2.71E-02* |
| **Protein** | **Protein measurement time point** | **Outcome measure time point** | **β-coefficient_adj_ (95% CI)** | **Adjusted p-value** |
| *DAS28* | | | | |
| EHD1 (Q9H4M9) | Baseline | 3 months | -0.01 (-1.28 – 1.26) | 0.99 |
| TCPH (Q99832) | Baseline | 3 months | 0.29 (-0.38 – 0.95)06 | 0.40 |
| CRP (P02741) | 3 months | 6 months | 0.03 (-0.20 – 0.26) | 0.82 |
| C9 (P02748) | 3 months | 6 months | 0.56 (-0.19 – 1.31) | 0.15 |
| *CRP measured using ELISA* | | | | |
| SELENOP (P49908) | Baseline | 6 months | 0.56 (-4.15 – 5.26) | 0.82 |
| MAP2K3 (P46734) | Baseline  3 months | 6 months  6 months | 0.82 (-7.09 – 8.73)  9.39 (0.44 – 18.33) | 0.84  4.83E-02* |
| CLTC (Q00610) | Baseline | 6 months | Not detected | N/A |
| SAA1 (P0DJI8) | 3 months | 6 months | -0.37 (-2.10 – 1.37) | 0.68 |
| MYLK (Q15746) | 3 months | 6 months | -7.91 (-16.86 – 1.03) | 0.09 |
| *VAS-GH* | | | | |
| ASPH (Q12797) | 3 months | 6 months | -3.19 (-25.64 – 19.25) | 0.78 |

Abbreviations: Aspartyl/asparaginyl β-hydroxylase (ASPH), clathrin heavy chain 1 (CLTC), complement component C9 (C9), C-reactive protein (CRP), Disease Activity Score of 28 Joints (DAS28), dual specificity mitogen-activated protein kinase kinase 3 (MAP2K3), EH domain-containing protein 1 (EHD1), enzyme-linked immunosorbent assay (ELISA), minimally clinically important difference (MCID), myosin light chain kinase, smooth muscle (MYLK), selenoprotein P (SELENOP), serum amyloid A-1 protein (SAA1), T-complex protein 1 subunit η (TCPH), visual analogue score of patient global health (VAS-GH).

UniProt identifiers are included in parentheses after each protein abbreviation.

*Supplementary Figure S1. Scatter plots demonstrating linearity assumption of numeric predictors from baseline protein and 3-month DAS28 remission multivariable model (logistic regression).*


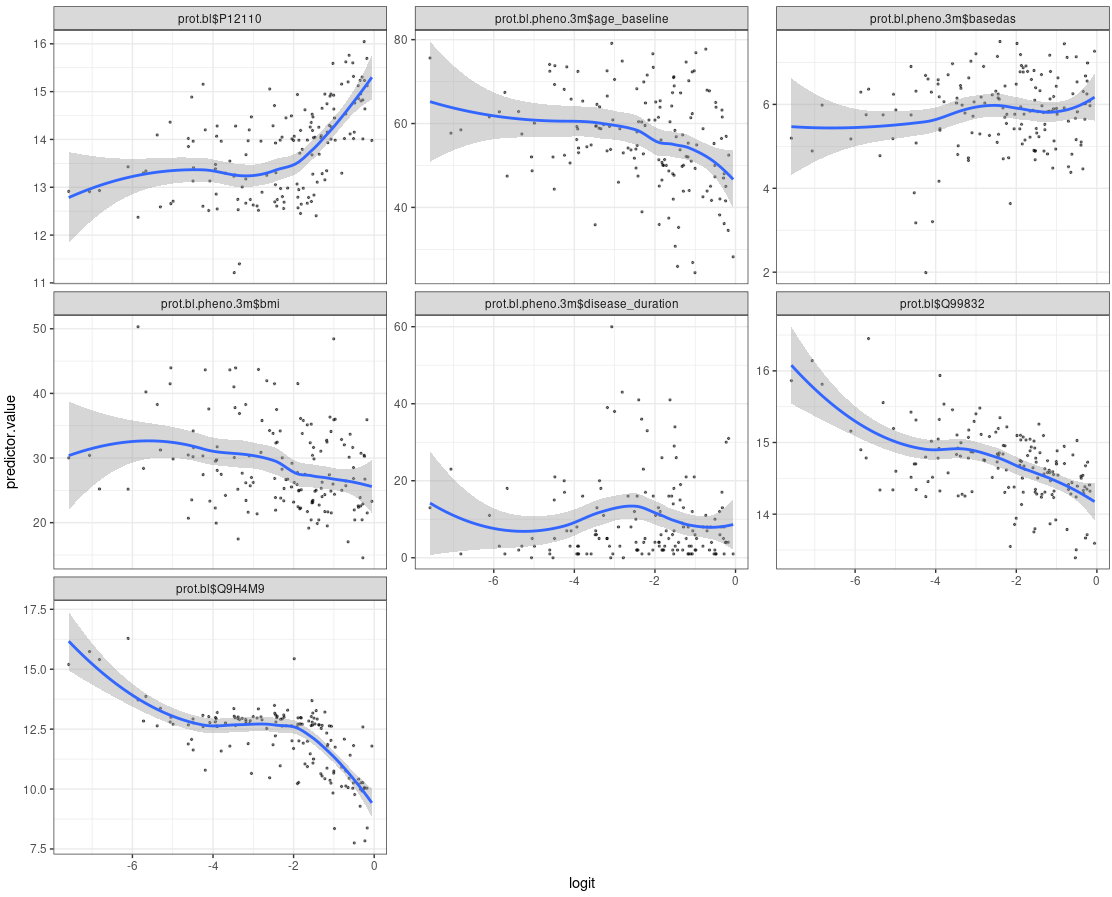


The smoothed scatter plots demonstrate linear relationships for the three proteins of interest: P12110, Q99832 and Q9H4M9. Other variables have been forced into the model to adjust for potential confounders.

*Supplementary Figure S2. Standardised residual plot from baseline protein and 3-month DAS28 remission multivariable model (logistic regression).*


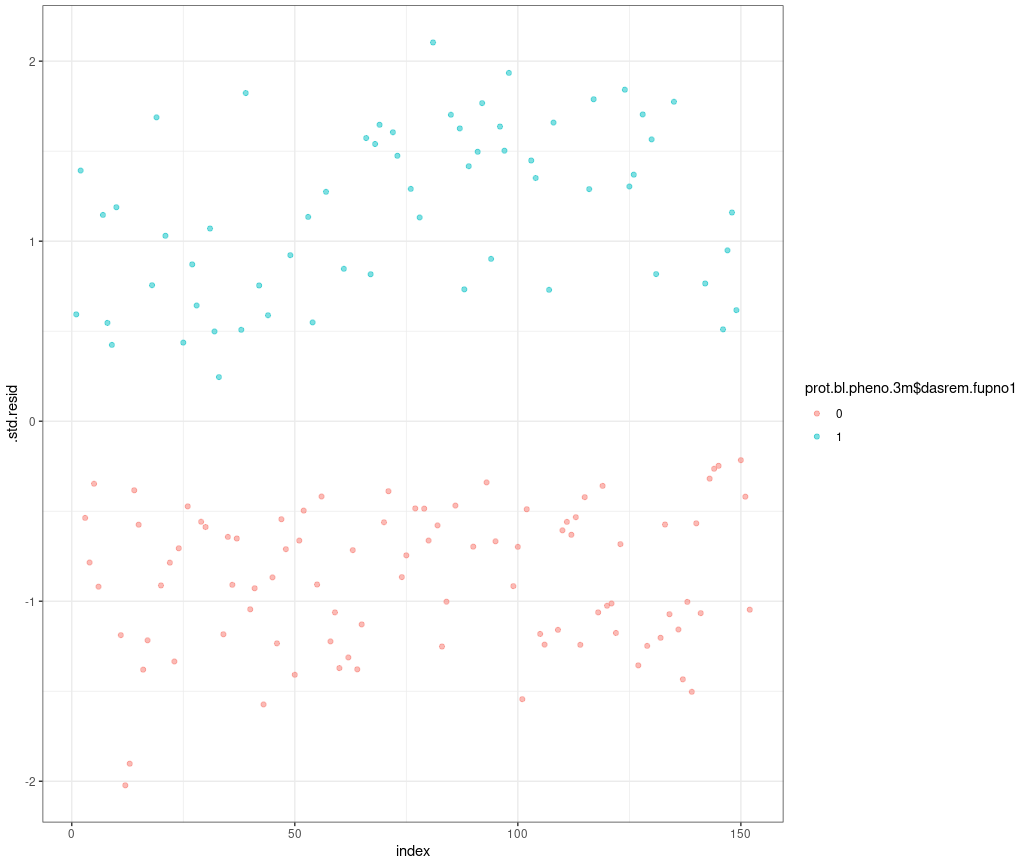


Points on the plot are clustered around low single digits on the y-axis without any clear patterns, indicating acceptable distribution of residuals.

*Supplementary Figure S3. Residuals vs fitted plot from baseline protein and 3-month DAS28 model (linear regression).*


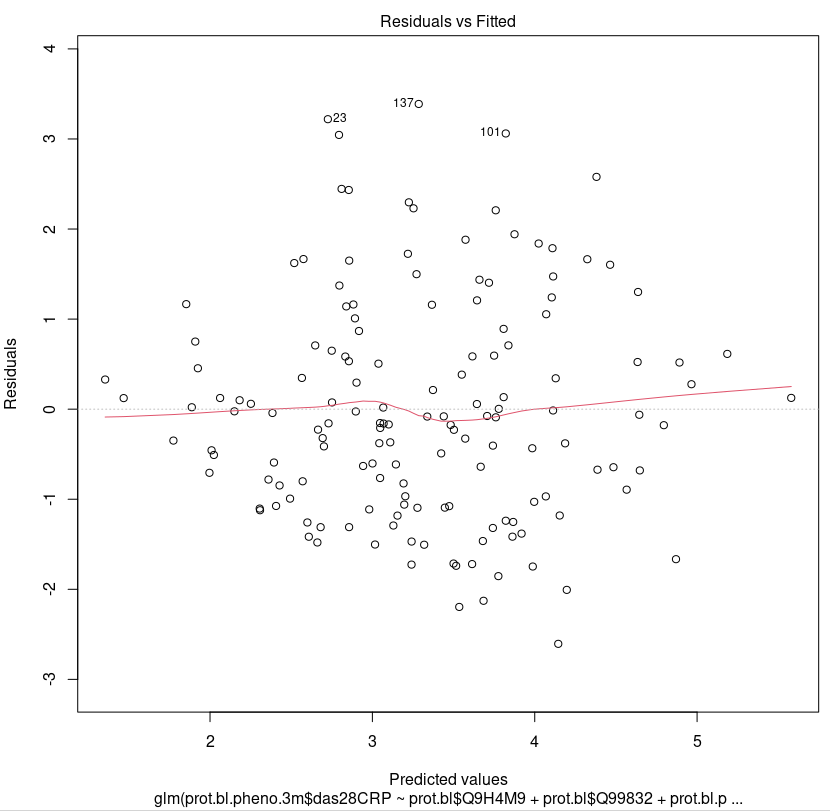


This demonstrates a largely horizontal line of best fit without distinct patterns of points, indicating a linear relationship between predictors and outcome variables.

*Supplementary Figure S4. Normal Q-Q plot from baseline protein and 3-month DAS28 model (linear regression).*


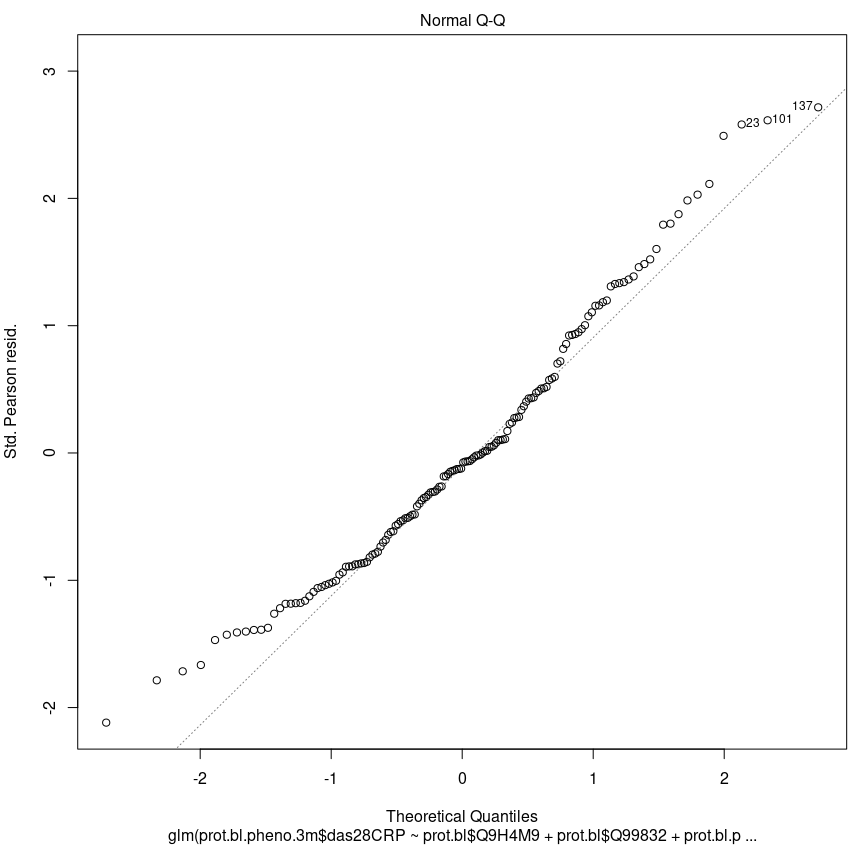


Residual points largely follow the straight dashed diagonal line, indicating that residuals are normally distributed.

*Supplementary Figure S5. Residuals vs fitted plot from baseline protein and 6-month CRP model (linear regression).*


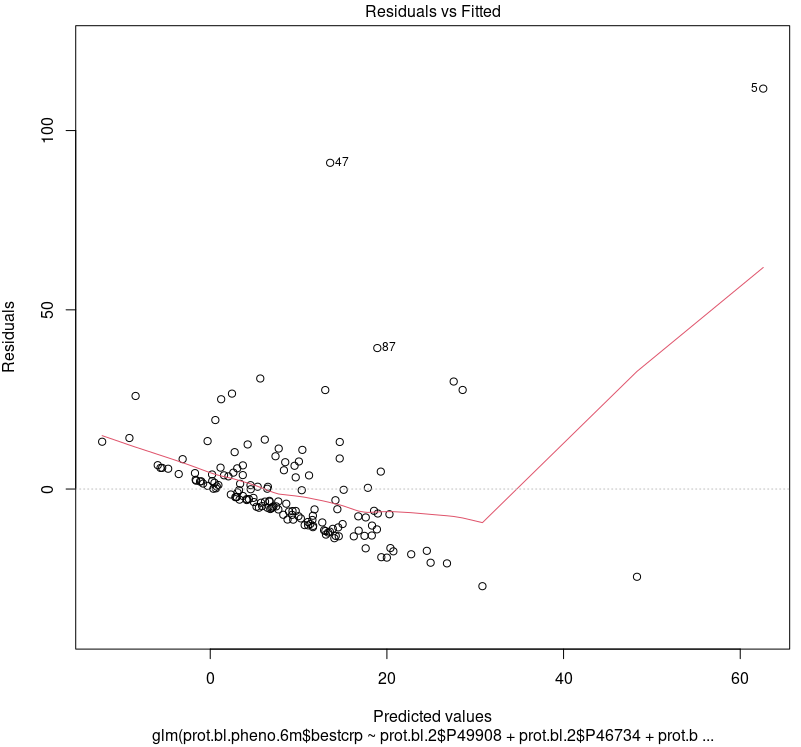


The red fit line would likely have remained horizontal if not for the outlier in the top right of the plot. This indicates a linear relationship in the model, apart from the extreme outliers.

*Supplementary Figure S6. Normal Q-Q plot from baseline protein and 3-month DAS28 model (linear regression).*


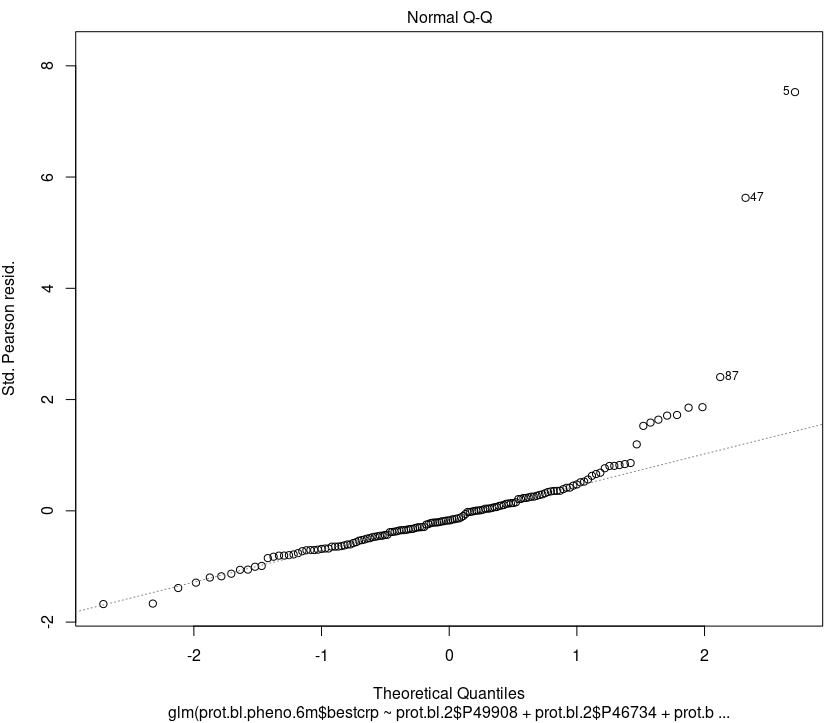


Residual points largely follow the straight dashed diagonal line, indicating that residuals are normally distributed, apart from some extreme outliers.

*Supplementary Figure S7. Residuals vs fitted plot from 3-month protein and 6-month VAS-GH model (linear regression).*

*
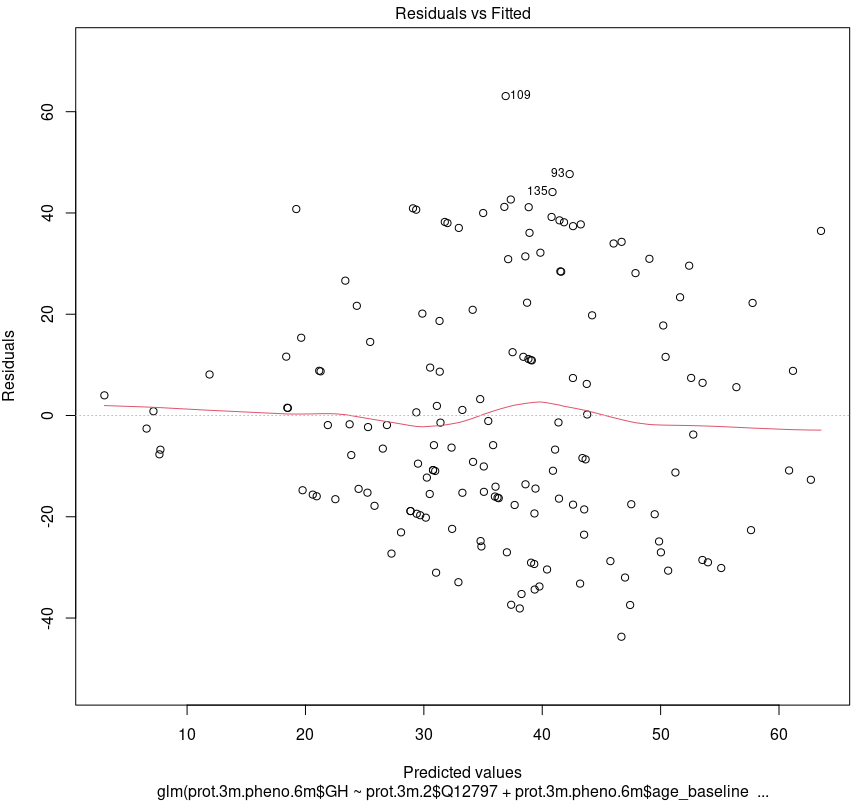
*

This demonstrates a largely horizontal line of best fit without distinct patterns of points, indicating a linear relationship between predictors and outcome variables.

*Supplementary Figure S8. Normal Q-Q plot from 3-month protein and 6-month VAS-GH model (linear regression).*


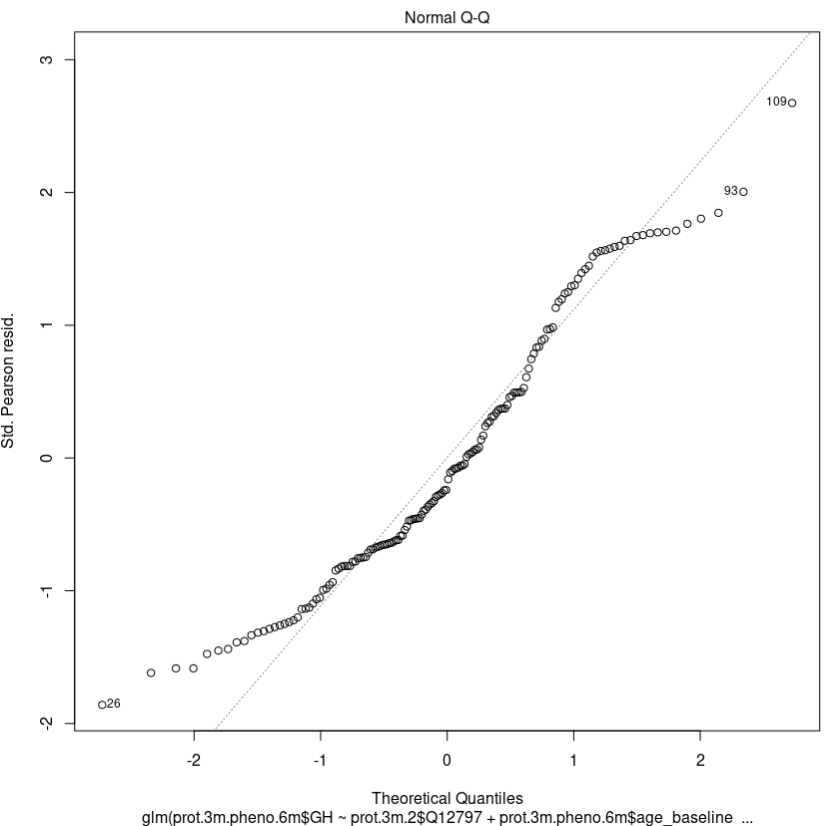


Residual points largely follow the straight dashed diagonal line, indicating that residuals are normally distributed.

*Supplementary Figure S9. Residuals vs fitted plot from 3-month protein and 6-month CRP model (linear regression).*


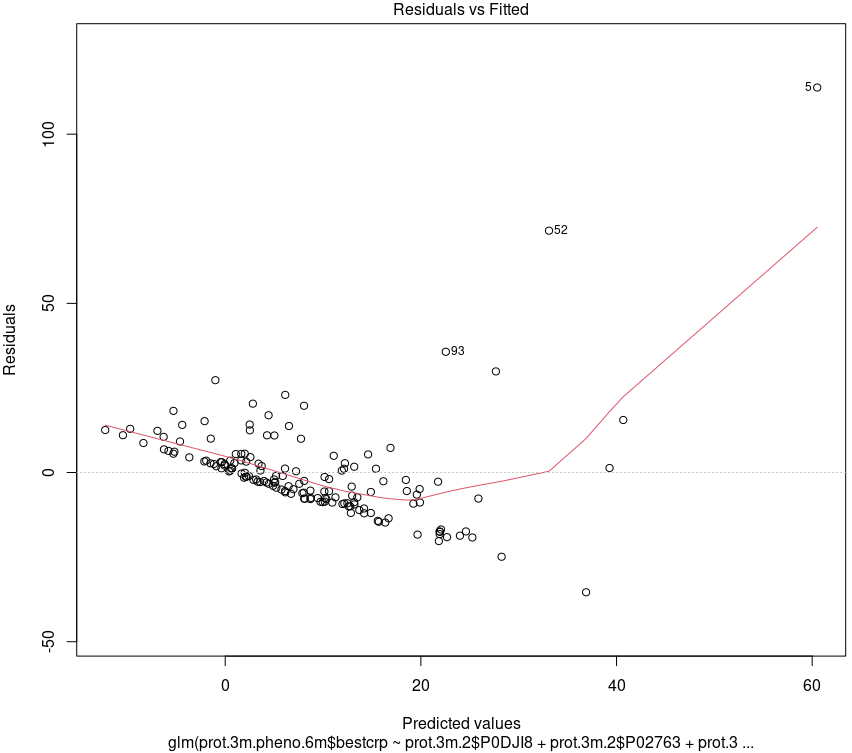


The red fit line would likely have remained horizontal if not for the outlier in the top right of the plot. This indicates a linear relationship in the model, apart from the extreme outliers.

*Supplementary Figure S10. Normal Q-Q plot from 3-month protein and 6-month CRP model (linear regression).*


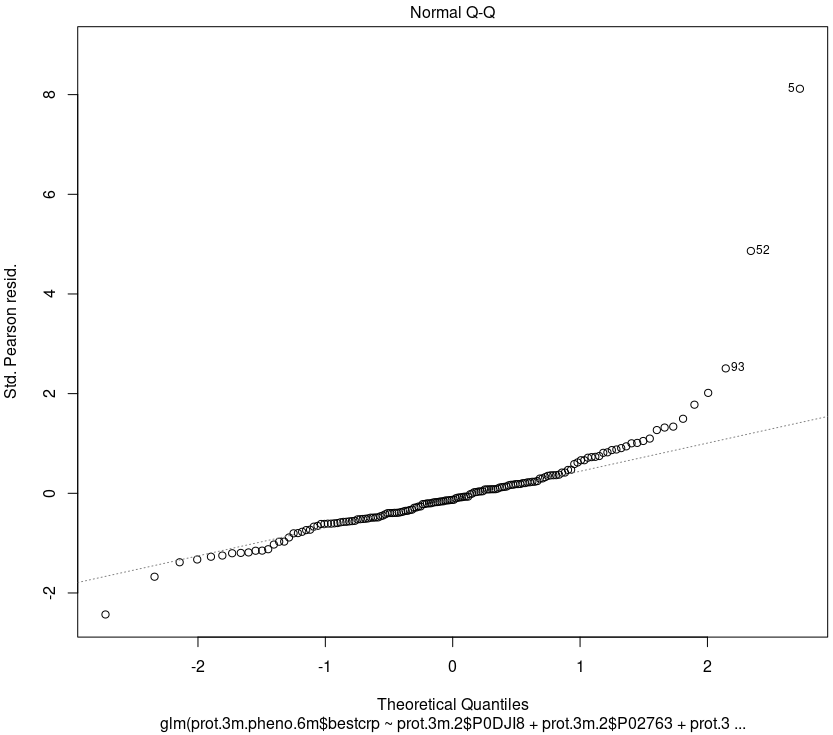


Residual points largely follow the straight dashed diagonal line, indicating that residuals are normally distributed, apart from some extreme outliers.

*Supplementary Figure S11. Residuals vs fitted plot from 3-month protein and 6-month CRP model (linear regression).*


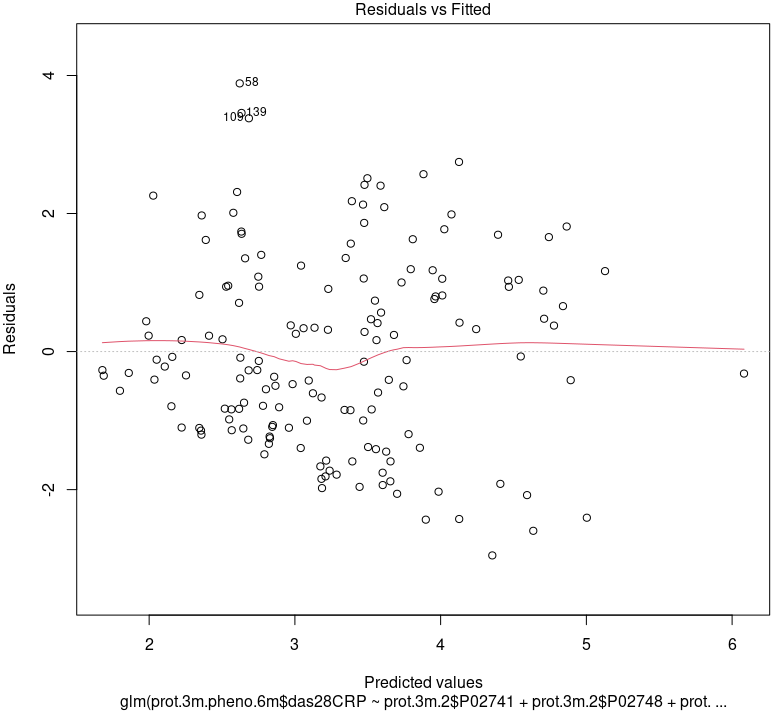


This demonstrates a largely horizontal line of best fit without distinct patterns of points, indicating a linear relationship between predictors and outcome variables.

*Supplementary Figure S12. Normal Q-Q plot from 3-month protein and 6-month CRP model (linear regression).*


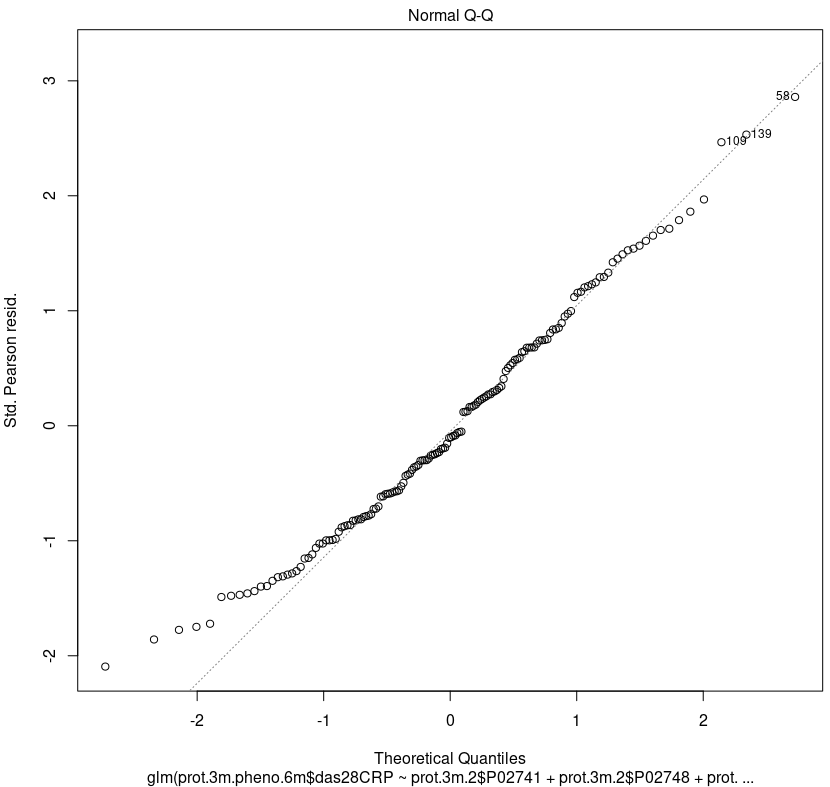


Residual points largely follow the straight dashed diagonal line, indicating that residuals are normally distributed.

*Supplementary Figure S13. Correlation between log2-transformed hsCRP measured using ELISA and CRP measured using SWATH-MS, baseline samples*.


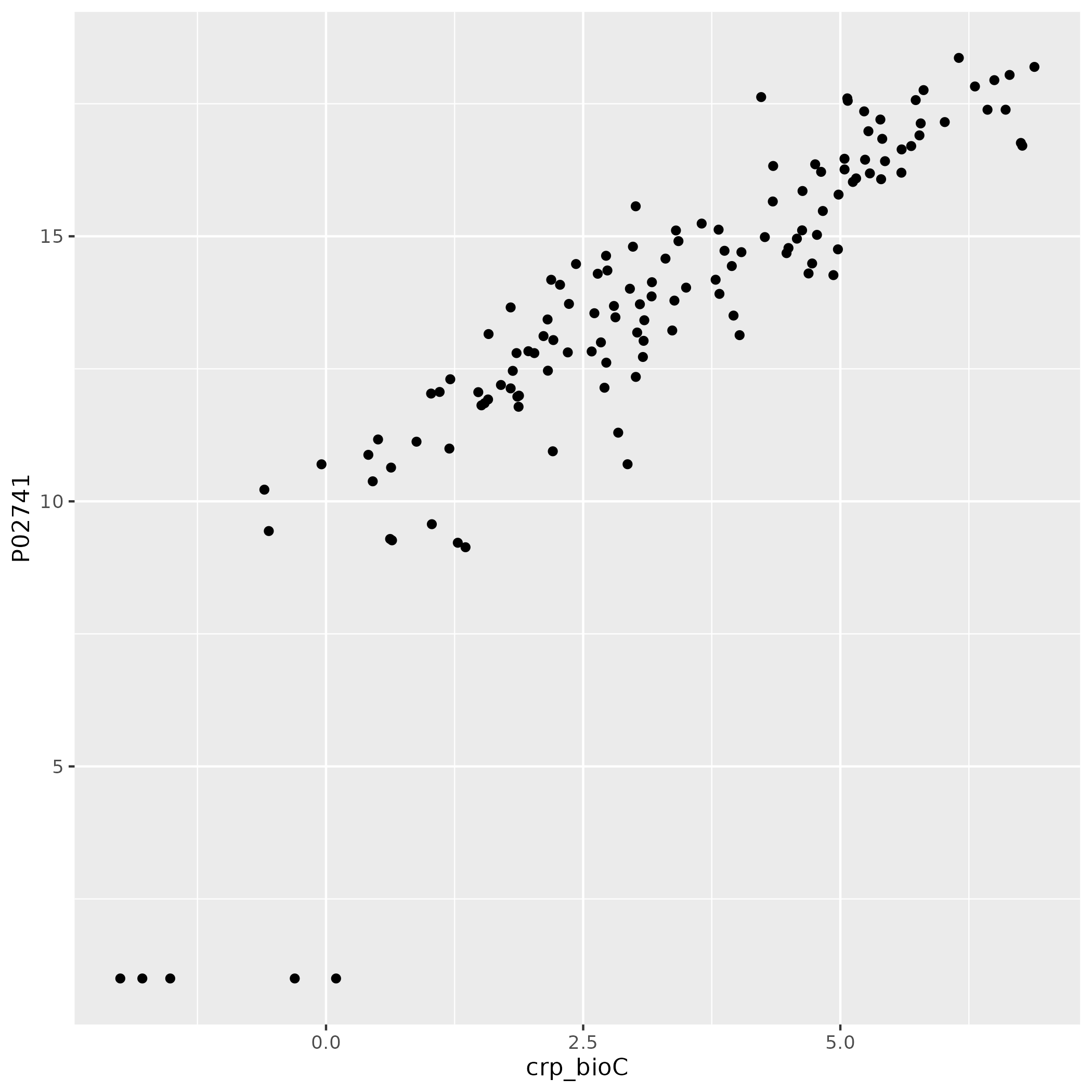


Pearson’s correlation coefficient 0.88, 95% CI 083. – 0.91, p-value = <2.2E-16.

*Supplementary Figure S14. Correlation between log2-transformed hsCRP measured using ELISA and CRP measured using SWATH-MS, 3-month samples.*


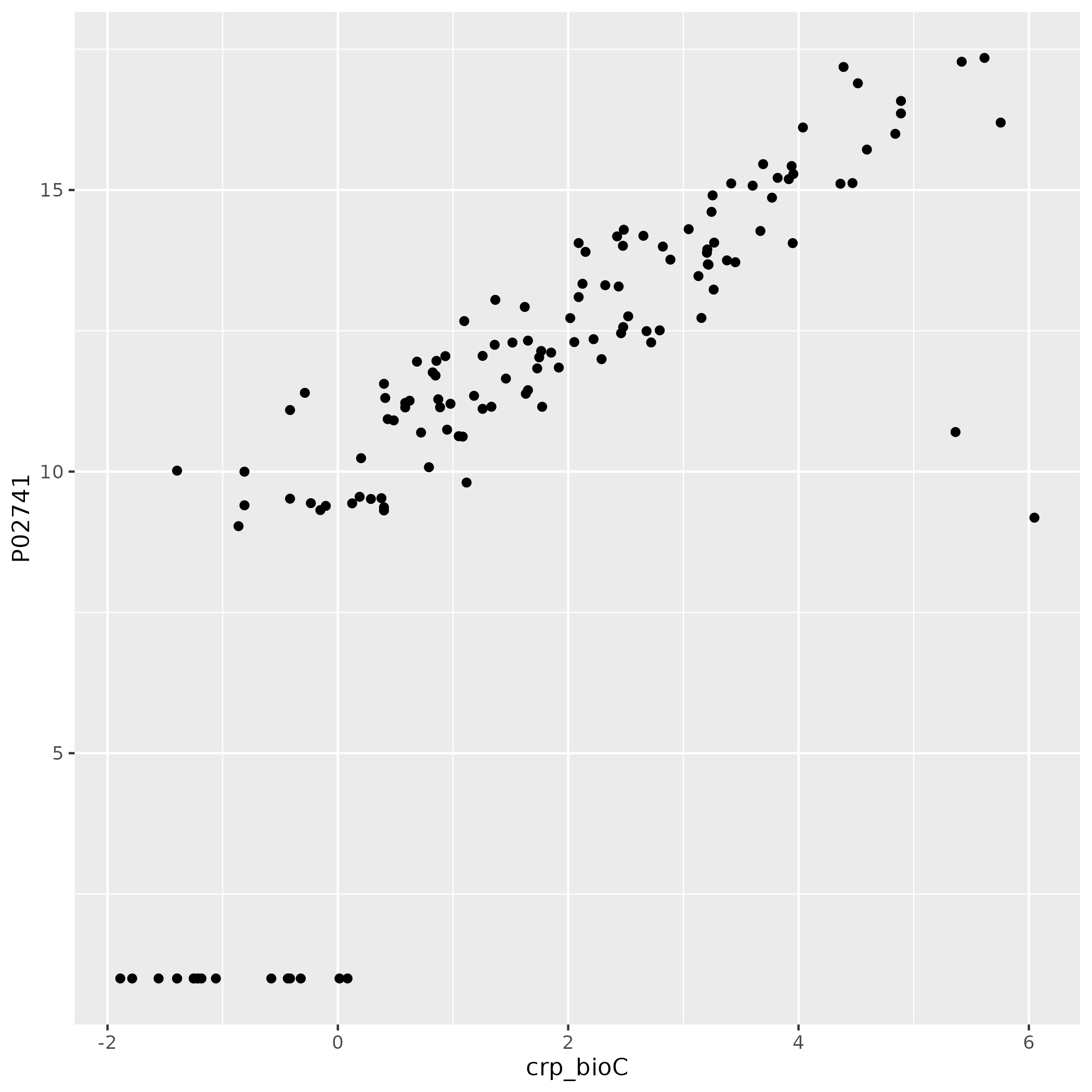


Pearson’s correlation coefficient 0.80, 95% CI 0.73 – 0.86, p-value = <2.2E-16.

**References**

1. UniProt [Available from: <https://www.uniprot.org.help/about>.

2. Kyoto Encyclopedia of Genes and Genomes.

3. Deutsch EW. Mass spectrometer output file format mzML. *Methods Mol Biol* 2010;604:319-31. doi: 10.1007/978-1-60761-444-9_22 [published Online First: 2009/12/17]

4. Rost HL, Rosenberger G, Navarro P, et al. OpenSWATH enables automated, targeted analysis of data-independent acquisition MS data. *Nat Biotechnol* 2014;32(3):219-23. doi: 10.1038/nbt.2841 [published Online First: 2014/04/15]

5. Liu Y, Buil A, Collins BC, et al. Quantitative variability of 342 plasma proteins in a human twin population. *Mol Syst Biol* 2015;11(1):786. doi: 10.15252/msb.20145728 [published Online First: 2015/02/06]

6. Teleman J, Rost HL, Rosenberger G, et al. DIANA--algorithmic improvements for analysis of data-independent acquisition MS data. *Bioinformatics* 2015;31(4):555-62. doi: 10.1093/bioinformatics/btu686 [published Online First: 2014/10/29]

7. MS Proteomics Tools [Available from: <https://github.com/msproteomicstools/msproteomicstools> accessed 9th September 2021.

8. Bioconductor [Available from: <https://www.bioconductor.org/> accessed 9th September 2021.

9. Choi M, Chang CY, Clough T, et al. MSstats: an R package for statistical analysis of quantitative mass spectrometry-based proteomic experiments. *Bioinformatics* 2014;30(17):2524-6. doi: 10.1093/bioinformatics/btu305 [published Online First: 2014/05/06]

10. Blattmann P, Heusel M, Aebersold R. SWATH2stats: An R/Bioconductor Package to Process and Convert Quantitative SWATH-MS Proteomics Data for Downstream Analysis Tools. *PLoS One* 2016;11(4):e0153160. doi: 10.1371/journal.pone.0153160 [published Online First: 2016/04/08]

11. Wickham H, Averick M, Bryan J, et al. Welcome to the tidyverse. *Journal of Open Source Software* 2019;4:1686-91. doi: <https://doi.org/0.21105/joss.01686>

12. Kuhn M. The caret Package 2019 [Available from: <https://topepo.github.io/caret/> accessed 9th September 2021.

13. Gu Z, Eils R, Schlesner M. Complex heatmaps reveal patterns and correlations in multidimensional genomic data. *Bioinformatics* 2016;32(18):2847-9. doi: 10.1093/bioinformatics/btw313 [published Online First: 2016/05/22]

14. Feng L, Moritz S, Nowak G, et al. imputeR: A general multivariate imputation framework. R package version 2.1. 2018 [Available from: <https://cran.r-project.org/web/packages/imputeR/index.html> accessed 9th September 2021.

15. Franzin A, Sambo F, Di Camillo B. bnstruct: an R package for Bayesian Network structure learning in the presence of missing data. *Bioinformatics* 2017;33(8):1250-52. doi: 10.1093/bioinformatics/btw807 [published Online First: 2016/12/23]

16. van Buuren S, Groothuis-Oudshoorn K. mice: Multivariate imputation by chained equations in R. *Journal of Statistical Software* 2011;45:1-67. doi: <https://doi.org/10.18637/jss.v045.i03>

17. Stekhoven DJ, Buhlmann P. MissForest--non-parametric missing value imputation for mixed-type data. *Bioinformatics* 2012;28(1):112-8. doi: 10.1093/bioinformatics/btr597 [published Online First: 2011/11/01]

18. Leek JT, Johnson WE, Parker HS, et al. sva: Surrogate variable analysis. R package version 3.40.0. 2021 [Available from: <https://bioconductor.org/packages/release/bioc/html/sva.html> accessed 9th September 2021.

19. Pawlowska J, Smolenska Z, Daca A, et al. Older age of rheumatoid arthritis onset is associated with higher activation status of peripheral blood CD4(+) T cells and disease activity. *Clin Exp Immunol* 2011;163(2):157-64. doi: 10.1111/j.1365-2249.2010.04294.x [published Online First: 2010/12/16]

20. Aletaha D, Maa JF, Chen S, et al. Effect of disease duration and prior disease-modifying antirheumatic drug use on treatment outcomes in patients with rheumatoid arthritis. *Ann Rheum Dis* 2019;78(12):1609-15. doi: 10.1136/annrheumdis-2018-214918 [published Online First: 2019/08/23]

21. Forslind K, Hafstrom I, Ahlmen M, et al. Sex: a major predictor of remission in early rheumatoid arthritis? *Ann Rheum Dis* 2007;66(1):46-52. doi: 10.1136/ard.2006.056937 [published Online First: 2006/12/13]

22. Smolen JS, Landewe R, Bijlsma J, et al. EULAR recommendations for the management of rheumatoid arthritis with synthetic and biological disease-modifying antirheumatic drugs: 2016 update. *Ann Rheum Dis* 2017;76(6):960-77. doi: 10.1136/annrheumdis-2016-210715 [published Online First: 2017/03/08]

23. Abuhelwa AY, Hopkins AM, Sorich MJ, et al. Association between obesity and remission in rheumatoid arthritis patients treated with disease-modifying anti-rheumatic drugs. *Sci Rep* 2020;10(1):18634. doi: 10.1038/s41598-020-75673-7 [published Online First: 2020/10/31]

24. Albrecht K, Zink A. Poor prognostic factors guiding treatment decisions in rheumatoid arthritis patients: a review of data from randomized clinical trials and cohort studies. *Arthritis Res Ther* 2017;19(1):68. doi: 10.1186/s13075-017-1266-4 [published Online First: 2017/03/25]

25. Taylor PC, Atzeni F, Balsa A, et al. The Key Comorbidities in Patients with Rheumatoid Arthritis: A Narrative Review. *J Clin Med* 2021;10(3) doi: 10.3390/jcm10030509 [published Online First: 2021/02/05]

26. Ghiassian SD, Menche J, Barabasi AL. A DIseAse MOdule Detection (DIAMOnD) algorithm derived from a systematic analysis of connectivity patterns of disease proteins in the human interactome. *PLoS Comput Biol* 2015;11(4):e1004120. doi: 10.1371/journal.pcbi.1004120 [published Online First: 2015/04/09]

27. Luck K, Kim DK, Lambourne L, et al. A reference map of the human binary protein interactome. *Nature* 2020;580(7803):402-08. doi: 10.1038/s41586-020-2188-x [published Online First: 2020/04/17]

28. Xie Z, Bailey A, Kuleshov MV, et al. Gene Set Knowledge Discovery with Enrichr. *Curr Protoc* 2021;1(3):e90. doi: 10.1002/cpz1.90 [published Online First: 2021/03/30]
